# Metabolic, inflammatory, and lipoprotein(a)-related risk profiling for incident ASCVD: a multi-biomarker study

**DOI:** 10.3389/fendo.2026.1901020

**Published:** 2026-08-14

**Authors:** Guanlin Chen, Yulong Lan, Dan Wu, Peifang Liu, Hong Zheng, Yuxian Wang, Youren Chen, Zekai Chen

**Affiliations:** 1 Department of Cardiology, Second Affiliated Hospital of Shantou University Medical College, Shantou, Guangdong, China; 2 Shaoguan University, Shaoguan, Guangdong, China

**Keywords:** atherosclerotic cardiovascular disease, coronary heart disease, high-sensitivity C-reactive protein, lipoprotein(a), stroke, triglyceride glucose index

## Abstract

**Aims:**

Metabolic dysfunction, inflammation, and lipoprotein(a) [Lp(a)] reflect different biomarker domains associated with atherosclerotic cardiovascular disease (ASCVD) risk. We examined the individual and joint associations of the triglyceride–glucose (TyG) index, high-sensitivity C-reactive protein (hs-CRP), and Lp(a) with incident ASCVD. Coronary heart disease (CHD) and stroke were analyzed as exploratory subtype outcomes of ASCVD.

**Methods:**

We included 320,506 UK Biobank participants free of ASCVD at baseline. TyG, hs-CRP, and Lp(a) were analyzed in quintiles, and elevated biomarkers were defined as values in the fifth quintile. Incident outcomes were assessed using multivariable-adjusted weighted Cox models.

**Results:**

Over a median follow-up of 13.88 years, 32,743 ASCVD events occurred. Compared with the lowest quintile, the highest quintile of TyG, hs-CRP, and Lp(a) was associated with higher ASCVD risk, with hazard ratios (HRs) of 1.32 (95% CI 1.27-1.38), 1.36 (1.30-1.43), and 1.23 (1.19-1.27), respectively. In exploratory analyses of ASCVD subtypes, the associations appeared generally more pronounced for CHD than for stroke. ASCVD risk increased progressively with the number of elevated biomarkers: HRs were 1.18 (1.15-1.21), 1.48 (1.42-1.53), and 1.85 (1.68-2.03) for one, two, and three elevated biomarkers, respectively. When the eight possible biomarker combinations were examined separately, concurrent elevation of TyG, hs-CRP, and Lp(a) identified the highest-risk profile for ASCVD (HR 1.90, 95% CI 1.71-2.11).

**Conclusions:**

TyG, hs-CRP, and Lp(a) provide complementary information for ASCVD risk stratification. Multi-biomarker profiling may help identify individuals with higher cardiometabolic risk than single-biomarker assessment alone.

## Introduction

Atherosclerotic cardiovascular disease (ASCVD) remains the leading cause of morbidity and mortality worldwide, despite substantial advances in lipid-lowering and preventive strategies (1). Although low-density lipoprotein cholesterol (LDL-C) is well established as a central causal factor in atherosclerosis (2), a substantial proportion of cardiovascular events still occur among individuals who achieve guideline-recommended LDL-C levels, underscoring the persistent burden of residual cardiovascular risk (3).

Beyond LDL-C, accumulating evidence indicates that lipoprotein(a) [Lp(a)] and systemic inflammation are important contributors to ASCVD risk (3, 4). Lp(a) is a genetically determined lipoprotein particle with pro-atherogenic and pro-thrombotic properties, largely independent of traditional lipid pathways and minimally modifiable through lifestyle interventions (5). Elevated Lp(a) levels have been consistently associated with incident coronary heart disease, stroke, and cardiovascular mortality across diverse populations (6, 7). In parallel, high-sensitivity C-reactive protein (hs-CRP), a marker of low-grade systemic inflammation, has been robustly associated with cardiovascular risk, even after adjustment for LDL-C and other conventional risk factors (8, 9). Large-scale cohort studies and randomized trials have further shown that dyslipidaemia and inflammation are each associated with ASCVD risk and may provide complementary risk information (10, 11).

However, within this combined risk framework, a critical biological axis has received comparatively less attention: metabolic dysfunction, particularly insulin resistance. Insulin resistance is increasingly recognized as a fundamental pathophysiological process underlying cardiometabolic diseases, contributing to endothelial dysfunction, chronic inflammation, dyslipidaemia, and the progression of atherosclerosis (12, 13). The triglyceride–glucose (TyG) index, calculated from fasting triglyceride and glucose concentrations, has emerged as a simple and reliable surrogate marker of insulin resistance (14). Numerous epidemiological studies have demonstrated that higher TyG index levels are strongly associated with increased risks of coronary heart disease, stroke, heart failure, and chronic kidney disease, even among individuals without overt diabetes (15–18).

Importantly, although LDL-C, Lp(a), and hs-CRP capture lipid burden, inherited lipoprotein risk, and inflammatory activity, respectively, they do not adequately reflect the metabolic–insulin resistance axis (4). Whether the TyG index provides additional ASCVD risk information beyond Lp(a) and hs-CRP remains largely unexplored (19). In particular, it remains unknown whether individuals with concomitant elevations in Lp(a), hs-CRP, and TyG index identify a subgroup at higher ASCVD risk than that defined by any single biomarker alone.

Leveraging the large-scale prospective UK Biobank cohort with linked ASCVD outcome data, we examined the individual and joint associations of Lp(a), hs-CRP, and the TyG index with incident ASCVD. Specifically, we sought to quantify the multivariable-adjusted associations of these biomarkers with ASCVD risk, evaluate whether their concurrent elevations identified individuals at higher risk, and assess the incremental predictive value of multi-biomarker profiling for ASCVD risk stratification. This multi-biomarker approach may improve characterization of ASCVD risk by integrating inherited lipoprotein, inflammatory, and metabolic biomarker domains.

## Methods

### Study design and population

#### The UK biobank

**Figure 1:**
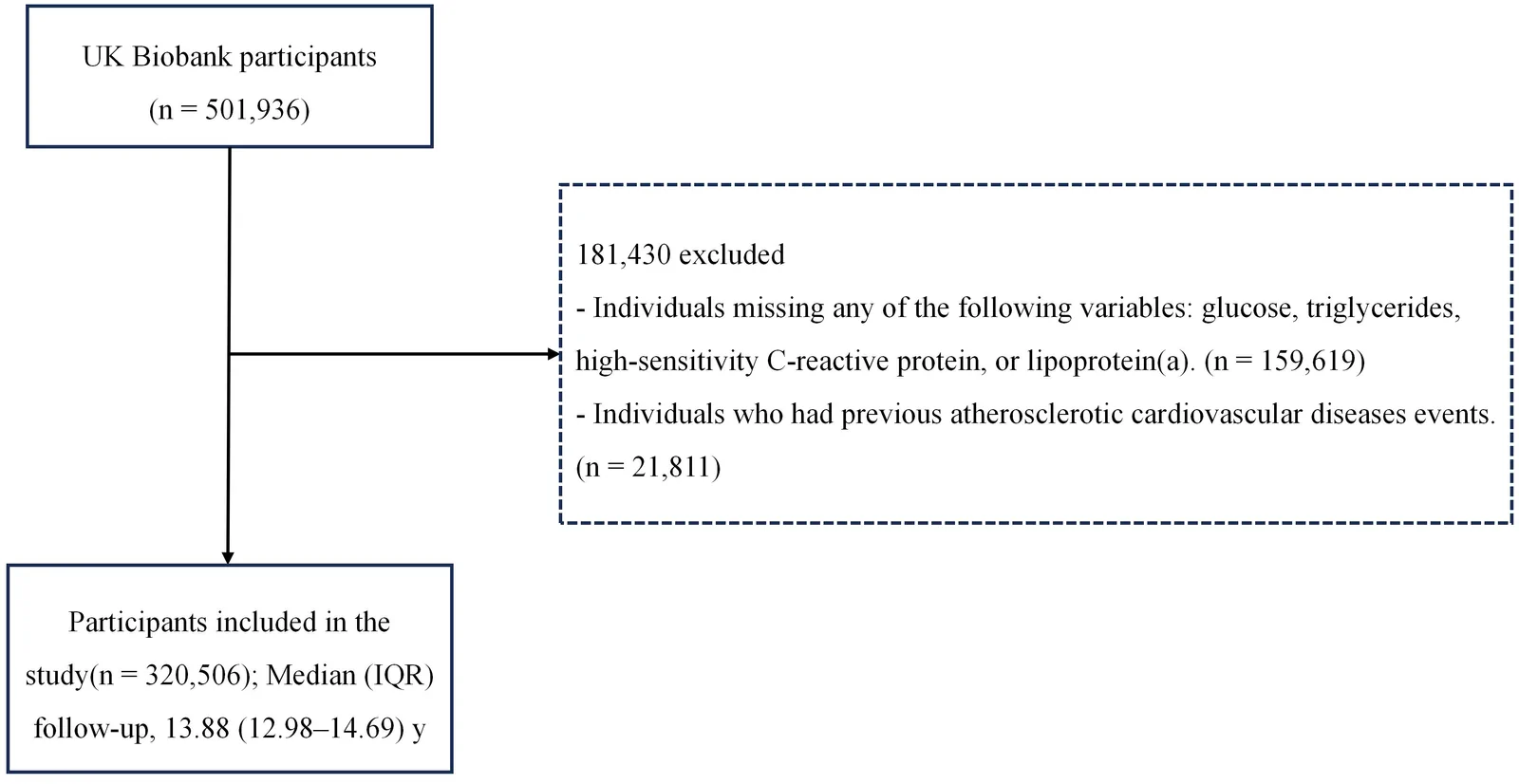
Study flowchart. The flowchart illustrates the participant selection process for the final analytical cohort after exclusion of individuals with missing biomarker data or prevalent ASCVD at baseline. IQR, interquartile range.

This study was conducted using data from the UK Biobank, a large-scale prospective observational cohort study (20, 21). A total of 501,936 participants were initially enrolled, and longitudinal follow-up data from baseline onward were analyzed. Participants were excluded if any of the following baseline measurements were missing: glucose (Glu), triglycerides (TG), hs-CRP, or Lp(a) (n = 159,619). In addition, individuals with pre-existing ASCVD at baseline were excluded (n = 21,811). The final analytical cohort comprised 320,506 participants, of whom 143,006 were men (44.62%) (**Figure 1**).

### Data collection

Baseline data on age, sex, Townsend Deprivation Index (TDI), ethnicity, current smoking status, physical activity level, and use of antihypertensive and lipid-lowering medications were collected using the touchscreen questionnaire. Physical activity was assessed using the International Physical Activity Questionnaire (IPAQ) (22), which captures the intensity, duration, and frequency of physical activity over the preceding seven days, and was quantified as metabolic equivalent task minutes per week (MET-min/week). TDI is a composite measure of socioeconomic deprivation derived from unemployment, non-car ownership, non-home ownership, and household overcrowding, with lower scores indicating higher socioeconomic status. Systolic and diastolic blood pressure were measured using a validated Omron HEM-7015IT digital sphygmomanometer or a standard manual sphygmomanometer, and the mean of two measurements was used for subsequent analyses. Body mass index (BMI) was calculated as weight in kilograms divided by height in meters squared (kg/m²).

Serum Glu, TG, hs-CRP, Lp(a), LDL-C, high-density lipoprotein cholesterol (HDL-C), total cholesterol (TC), and creatinine were measured in a central laboratory using the Beckman Coulter AU5800 clinical chemistry analyzer. Glycated hemoglobin (HbA1c) was determined by high-performance liquid chromatography using the Bio-Rad VARIANT II Turbo system. The TyG index was calculated from baseline triglyceride and glucose concentrations using the formula ln [triglyceride (mg/dL) × glucose (mg/dL)/2] (15, 16). Because fasting before blood collection was not uniformly required in the UK Biobank, the TyG index should be interpreted as a baseline biochemistry-derived measure obtained under non-standardized fasting conditions. All laboratory parameters and derived variables were based on measurements obtained at the baseline visit. Detailed information on laboratory assays and measurement procedures is available in the UK Biobank online study protocol.

### Atherosclerotic cardiovascular diseases

The primary outcome of this study was incident ASCVD, defined as the first occurrence of coronary heart disease (CHD) or stroke. ASCVD events were ascertained using codes from the International Classification of Diseases, 10th Revision (ICD-10), with CHD identified using codes I20–I25 and stroke identified using codes I60–I64.

The end of follow-up was defined as the earliest occurrence of the assessment center follow-up end date, the date of first incident ASCVD, or death, whichever occurred first. Follow-up commenced on the date of completion of the baseline assessment. Participants lost to follow-up were censored at the date of the last available follow-up information, with follow-up time calculated from the baseline assessment date to the censoring date.

### Statistical analysis

Baseline characteristics were compared across the entire cohort and according to biomarker quintiles. Normally distributed continuous variables were presented as mean ± standard deviation (SD) and compared using one-way analysis of variance (ANOVA). Non-normally distributed continuous variables were summarized as median with interquartile range (Q1–Q3) and compared using the Kruskal–Wallis test. The normality of continuous variables was assessed using the Kolmogorov–Smirnov test. Categorical variables were reported as counts and percentages, and differences between groups were assessed using the chi-square test.

Extreme biomarker values were handled using winsorization, with cut-off points set at the 0.1st and 99.9th percentiles. Missing covariate data were addressed using univariate deterministic imputation. For continuous variables, mean imputation was applied for approximately normally distributed variables, whereas median imputation was used for non-normally distributed variables; for categorical variables, the mode was imputed. Restricted cubic spline (RCS) regression models were employed to examine potential dose–response relationships between the three biomarkers and the risk of ASCVD and its subtypes. The proportional hazards assumption was assessed using Schoenfeld residuals (**Supplementary Table 1** and **Supplementary Figure 1**). For categorical biomarker exposures, propensity score-based inverse probability weighting Cox regression models were used to estimate associations with incident outcomes while improving covariate balance across exposure categories. Multinomial logistic regression was used to estimate the probability of exposure-category membership conditional on baseline covariates. Stabilized inverse probability weights were calculated as the marginal probability of the observed exposure category divided by the corresponding conditional probability from the propensity score model. Stabilized weights were truncated at the 1st and 99th percentiles to reduce the influence of extreme weights and were incorporated into weighted Cox models with robust sandwich standard errors.

The primary analyses focused on incident ASCVD as the main outcome. Analyses of ASCVD subtypes, including CHD and stroke, were considered exploratory. Although these analyses generally showed directionally consistent associations, they should be interpreted with caution because no formal adjustment for multiple comparisons was performed. In the primary analyses, participants were categorized into quintiles according to biomarker levels, with the first and fifth quintiles representing the lowest and highest levels, respectively. The first quintile served as the reference group, and HRs with 95% CIs for ASCVD were estimated for the second through fifth quintiles. Model 1 was adjusted for age and sex. Model 2 was further adjusted for TDI, ethnicity, current smoking status, physical activity, systolic blood pressure, BMI, creatinine, and use of antihypertensive and lipid-lowering medications. Model 3 was additionally adjusted for the other two biomarkers (TyG index, hs-CRP, and Lp(a), as applicable) based on Model 2.

**Figure 2:**
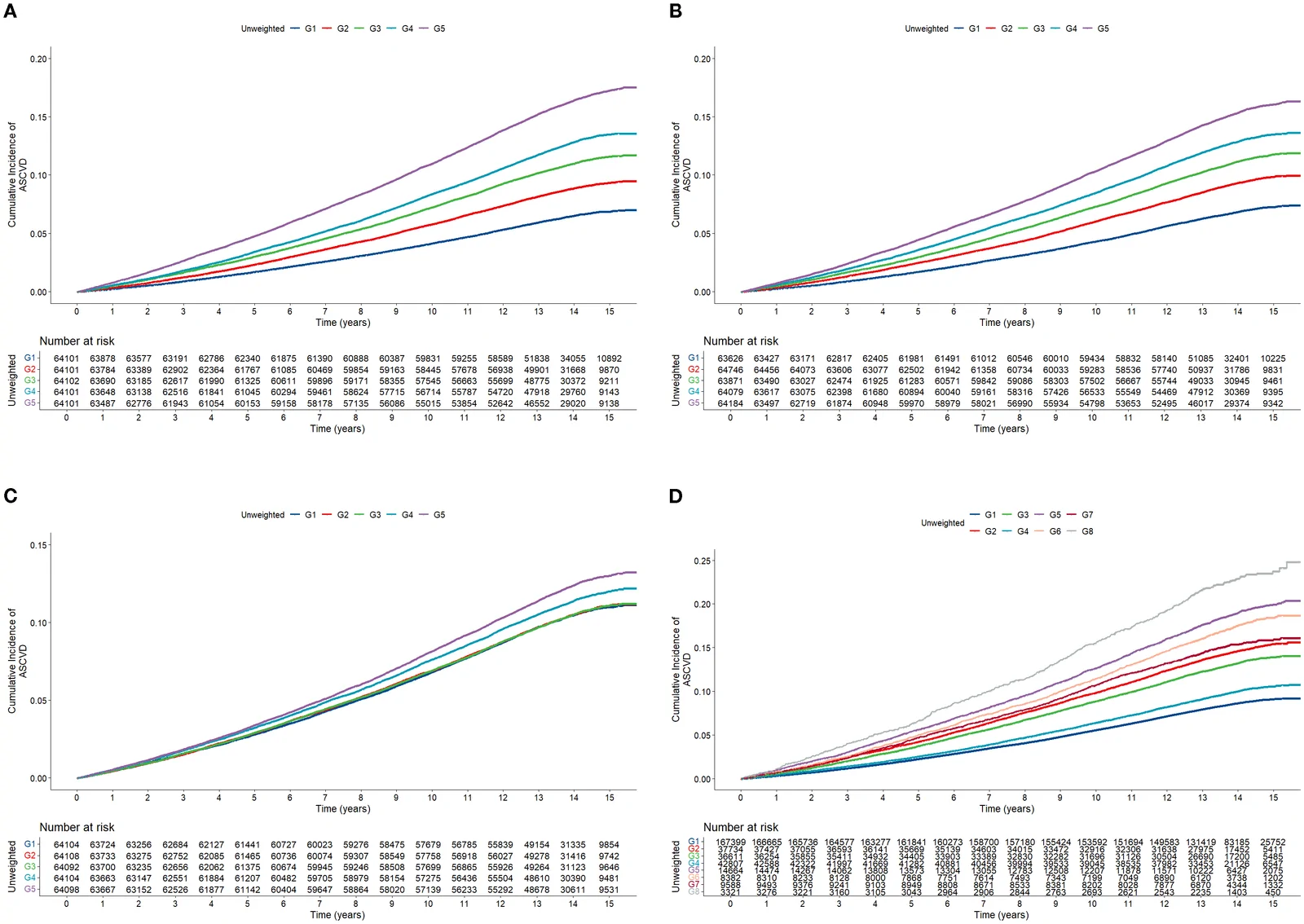
Cumulative incidence of atherosclerotic cardiovascular disease according to increasing quintiles of TyG, hs-CRP, and Lp(a). **(A)** According to increasing quintiles of TyG. **(B)** According to increasing quintiles of hs-CRP. **(C)** According to increasing quintiles of Lp(a). **(D)** According to according to the combined biomarker pattern. **(D)** G1, TyG, hs-CRP, and Lp(a) all below the fifth quintile; G2, TyG in the fifth quintile only; G3, hs-CRP in the fifth quintile only; G4, Lp(a) in the fifth quintile only; G5, TyG and hs-CRP in the fifth quintile; G6, TyG and Lp(a) in the fifth quintile; G7, hs-CRP and Lp(a) in the fifth quintile; and G8, TyG, hs-CRP, and Lp(a) all in the fifth quintile. TyG, triglyceride–glucose; hs-CRP, high-sensitivity C-reactive protein; Lp(a), lipoprotein(a).

To evaluate the joint associations of the three biomarkers, participants were classified based on whether each biomarker was in the fifth quintile, resulting in four groups with 0, 1, 2, or 3 biomarkers in the highest quintile. Building on this classification, an eight-level categorical variable was constructed to characterize distinct biomarker combination patterns (1): TyG, hs-CRP, and Lp(a) all below the fifth quintile (2); TyG in the fifth quintile only (3); hs-CRP in the fifth quintile only (4); Lp(a) in the fifth quintile only (5); TyG and hs-CRP in the fifth quintile (6); TyG and Lp(a) in the fifth quintile (7); hs-CRP and Lp(a) in the fifth quintile; and (8) TyG, hs-CRP, and Lp(a) all in the fifth quintile. In the analyses, participants with 0 biomarkers in the fifth quintile and those in category 1 were separately used as reference groups to estimate associations between the remaining groups and the risk of ASCVD. Model 1 was adjusted for age and sex, whereas Model 2 was further adjusted for TDI, ethnicity, current smoking status, physical activity, systolic blood pressure, BMI, creatinine, and use of antihypertensive and lipid-lowering medications. Kaplan–Meier curves were generated to compare cumulative incidence across biomarker quintile groups and across the combined biomarker patterns (**Figure 2**).

Sensitivity and subgroup analyses were restricted to the primary ASCVD outcome. Exploratory subgroup analyses were conducted by sex and age group, and multiplicative interactions were assessed using cross-product terms between biomarker categories and subgroup variables. No formal adjustment for multiple comparisons was applied to exploratory subtype or subgroup analyses; therefore, the corresponding *P* values, including *P* values for interaction, were considered nominal and hypothesis-generating. A series of sensitivity analyses were performed to assess the robustness of the findings. First, to minimize potential reverse causation, participants who experienced outcome events within the first two years of follow-up were excluded. Second, participants with a diagnosis of malignancy at baseline were excluded, given the potential influence of malignant tumors on metabolic status. Because weighting does not resolve potential violations of the proportional hazards assumption, we performed follow-up interval-specific Cox analyses, estimating HRs separately for 0-5, 5-10, and >10 years of follow-up. For sensitivity analyses using alternative biomarker thresholds, elevated TyG was defined as ≥8.67, a data-derived threshold corresponding to the point at which the restricted cubic spline-estimated HR equaled 1.0, given the absence of a universally accepted clinical cutoff. Elevated hs-CRP and Lp(a) were defined using clinically relevant thresholds of ≥2.0 mg/L and >105 nmol/L, respectively (5, 23). The combined biomarker profiles were then reconstructed, and the analysis was repeated for incident ASCVD. Finally, competing risk regression (Fine–Gray model) was applied to account for the potential competing risk of death.

To evaluate the incremental predictive value of TyG, hs-CRP, and Lp(a) for 10-year incident ASCVD, we compared a clinical reference model with four biomarker-augmented models that additionally included TyG, hs-CRP, Lp(a), or all three biomarkers simultaneously. The clinical reference model included age, sex, ethnicity, Townsend Deprivation Index, smoking status, physical activity, systolic blood pressure, body mass index, total cholesterol, creatinine, antihypertensive medication use, and lipid-lowering medication use. TyG, hs-CRP, and Lp(a) were modelled as standardized continuous variables. hs-CRP and Lp(a) were log-transformed before standardization. Incremental predictive performance was assessed using Harrell’s C-statistic, 10-year time-dependent AUC, Brier score, continuous net reclassification improvement and integrated discrimination improvement. Each biomarker-augmented model was compared with the clinical reference model. These analyses were restricted to the primary ASCVD outcome.

All statistical analyses were performed using R software (version 4.4.0), and a two-sided *P* value < 0.05 was considered statistically significant.

## Results

### Baseline characteristics

Baseline characteristics of the study participants stratified by quintiles of TyG, hs-CRP, and Lp(a) at enrollment are shown in **Table 1** and **Supplementary Table 2**. Among the 320,506 participants, the mean age was 56.17 years, and 44.62% were men. Overall, 10.27% and 12.60% of participants reported use of antihypertensive and lipid-lowering medications, respectively. At baseline, the mean TyG index was 8.70, whereas the median hs-CRP and Lp(a) concentrations were 1.31 (0.65–2.73) mg/L and 20.99 (9.55–61.50) nmol/L, respectively.

**Table 1:** Baseline characteristics of TDI, TC, LDL-C, HbA1c and creatinine among the study participants according to increasing quintiles of TyG, hs-CRP, and Lp(a).

Characteristic	Quintile of Baseline TyG				
	Quintile 1	Quintile 2	Quintile 3	Quintile 4	Quintile 5
	N = 64,101	N = 64,101	N = 64,102	N = 64,101	N = 64,101
Range	6.80–8.22	8.22–8.53	8.53–8.82	8.82–9.16	9.16–11.68
TDI	-1.34 ± 3.09	-1.43 ± 3.02	-1.41 ± 3.02	-1.39 ± 3.05	-1.19 ± 3.12
TC, mmol/l	5.34 ± 0.96	5.63 ± 1.00	5.78 ± 1.05	5.94 ± 1.11	6.09 ± 1.23
LDL-C, mmol/l	3.20 ± 0.70	3.50 ± 0.75	3.67 ± 0.80	3.81 ± 0.85	3.86 ± 0.92
HbA1c, mmol/mol	34.05 ± 4.39	34.80 ± 4.40	35.37 ± 4.67	36.12 ± 5.29	38.69 ± 9.80
Creatinine, umol/L	68.51 ± 14.58	70.47 ± 16.25	71.86 ± 15.28	73.35 ± 18.33	74.68 ± 20.06
Quintile of Baseline hs-CRP					
	Quintile 1	Quintile 2	Quintile 3	Quintile 4	Quintile 5
	N = 63,626	N = 64,746	N = 63,871	N = 64,079	N = 64,184
Range, mg/L	0.10–0.55	0.55–1.00	1.00–1.73	1.73–3.29	3.29–52.14
TDI	-1.54 ± 2.96	-1.55 ± 2.96	-1.46 ± 3.01	-1.29 ± 3.09	-0.93 ± 3.24
TC, mmol/l	5.62 ± 1.04	5.74 ± 1.08	5.81 ± 1.10	5.85 ± 1.13	5.76 ± 1.15
LDL-C, mmol/l	3.45 ± 0.79	3.58 ± 0.82	3.65 ± 0.84	3.70 ± 0.86	3.66 ± 0.87
HbA1c, mmol/mol	34.43 ± 4.98	35.07 ± 5.35	35.67 ± 5.83	36.32 ± 6.45	37.53 ± 7.90
Creatinine, umol/L	70.37 ± 14.37	72.07 ± 15.04	72.57 ± 16.09	72.29 ± 17.11	71.57 ± 22.00
Quintile of Baseline Lp(a)					
	Quintile 1	Quintile 2	Quintile 3	Quintile 4	Quintile 5
	N = 64,104	N = 64,108	N = 64,092	N = 64,104	N = 64,098
Range, nmol/L	3.80–8.18	8.18–14.84	14.84–31.85	31.85–81.21	81.21–188.40
TDI	-1.41 ± 3.01	-1.43 ± 3.01	-1.41 ± 3.02	-1.20 ± 3.16	-1.31 ± 3.10
TC, mmol/l	5.63 ± 1.10	5.68 ± 1.08	5.79 ± 1.09	5.84 ± 1.13	5.83 ± 1.10
LDL-C, mmol/l	3.49 ± 0.84	3.54 ± 0.82	3.64 ± 0.83	3.68 ± 0.86	3.68 ± 0.83
HbA1c, mmol/mol	35.85 ± 6.65	35.68 ± 6.39	35.73 ± 5.92	35.94 ± 6.18	35.83 ± 6.23
Creatinine, umol/L	71.70 ± 15.93	71.88 ± 16.69	71.62 ± 16.04	71.66 ± 19.03	72.01 ± 17.89

TyG, triglyceride–glucose; hs-CRP, high-sensitivity C-reactive protein; Lp(a), lipoprotein(a); TDI, Townsend Deprivation Index; TC, total cholesterol; LDL-C, low-density lipoprotein cholesterol; HbA1c, glycated hemoglobin.

### Associations of TyG, hs-CRP, and Lp(a) concentrations with ASCVD events and their subtypes

The median follow-up duration was 13.88 years (interquartile range [IQR], 12.98–14.69). Among the 320,506 participants, a total of 32,743 incident ASCVD events were documented, including 12,801 events among women and 19,942 among men. The cumulative incidence of ASCVD shown in **Figure 2**. With increasing quintiles, the associations of TyG and hs-CRP with risk increased progressively. In contrast, the association between Lp(a) and risk became more apparent primarily among individuals in the fourth and fifth quintiles, corresponding to concentrations above 31.85 nmol/L. Compared with participants in the first quintile, those in the fifth quintile had higher multivariable-adjusted risks of ASCVD, with HRs of 1.32 (95% CI, 1.27–1.38) for TyG, 1.36 (95% CI, 1.30–1.43) for hs-CRP, and 1.23 (95% CI, 1.19–1.27) for Lp(a).

**Figure 3:**
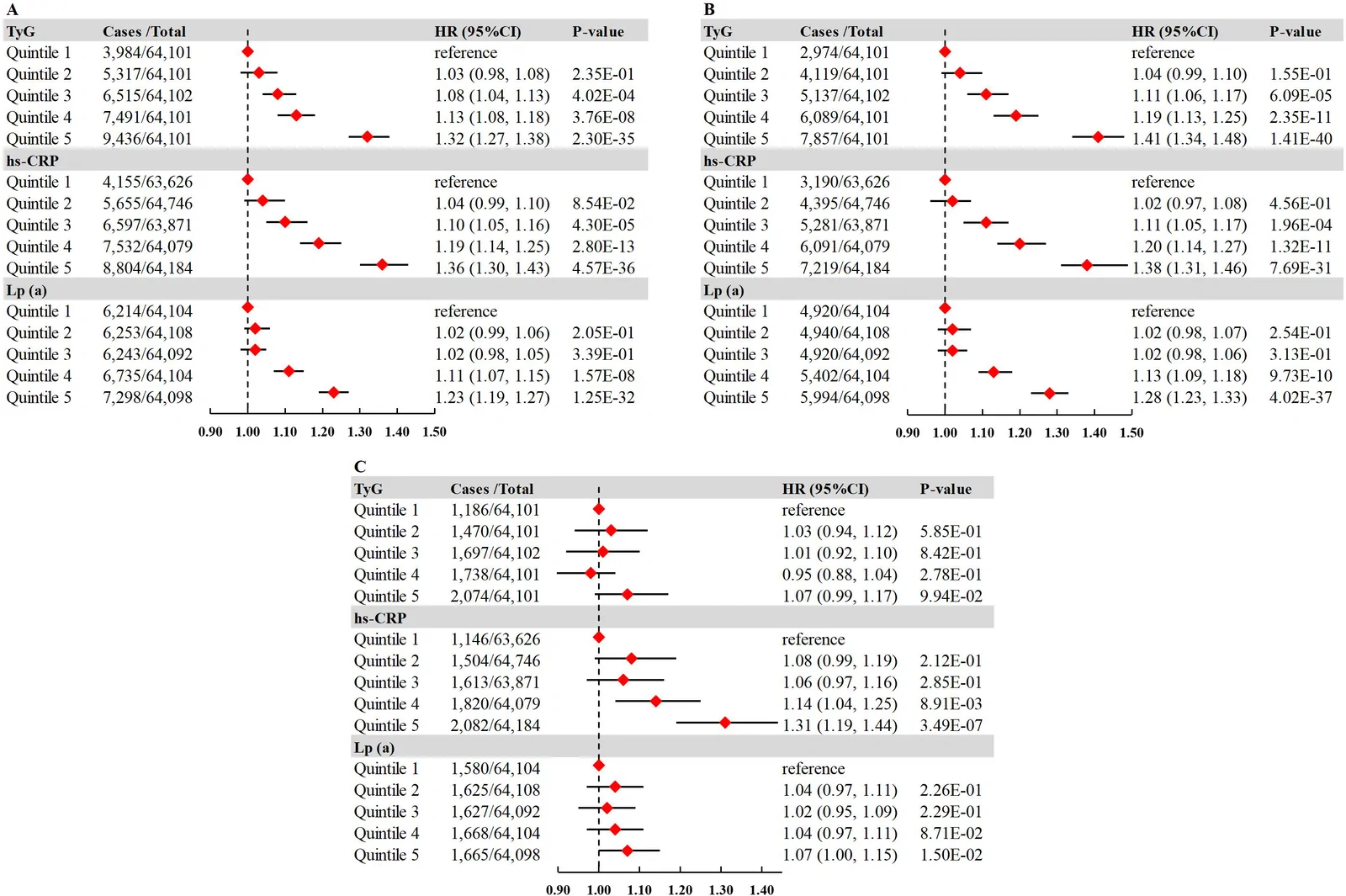
Associations of TyG, hs-CRP, and Lp(a) concentrations with ASCVD events and their subtypes. **(A)** Associations of TyG, hs-CRP, and Lp(a) concentrations with ASCVD events; **(B)** Associations of TyG, hs-CRP, and Lp(a) concentrations with CHD events; **(C)** Associations of TyG, hs-CRP, and Lp(a) concentrations with stroke events. Model was adjusted for age, sex, TDI, ethnicity, current smoking status, physical activity, systolic blood pressure, BMI, creatinine, use of antihypertensive and lipid-lowering medications, and the other two biomarkers (TyG index, hs-CRP, and Lp(a), as applicable). TyG, triglyceride–glucose; hs-CRP, high-sensitivity C-reactive protein; Lp(a), lipoprotein(a); ASCVD, atherosclerotic cardiovascular disease; CHD, coronary heart disease, TDI, Townsend deprivation index; BMI, body mass index; HR, hazard ratio; CI, confidence interval.

In exploratory subtype analyses of ASCVD subtypes, the associations appeared more pronounced for CHD than for stroke. The multivariable-adjusted HRs for CHD comparing the fifth with the first quintile were 1.41 (95% CI, 1.34-1.48) for TyG, 1.38 (95% CI, 1.31-1.46) for hs-CRP, and 1.28 (95% CI, 1.23-1.33) for Lp(a). Correspondingly, the HRs for stroke were 1.07 (95% CI, 0.99-1.17), 1.31 (95% CI, 1.19-1.44), and 1.07 (95% CI, 1.00-1.15), respectively (**Figure 3** and **Supplementary Table 4**).

**Figure 4:**
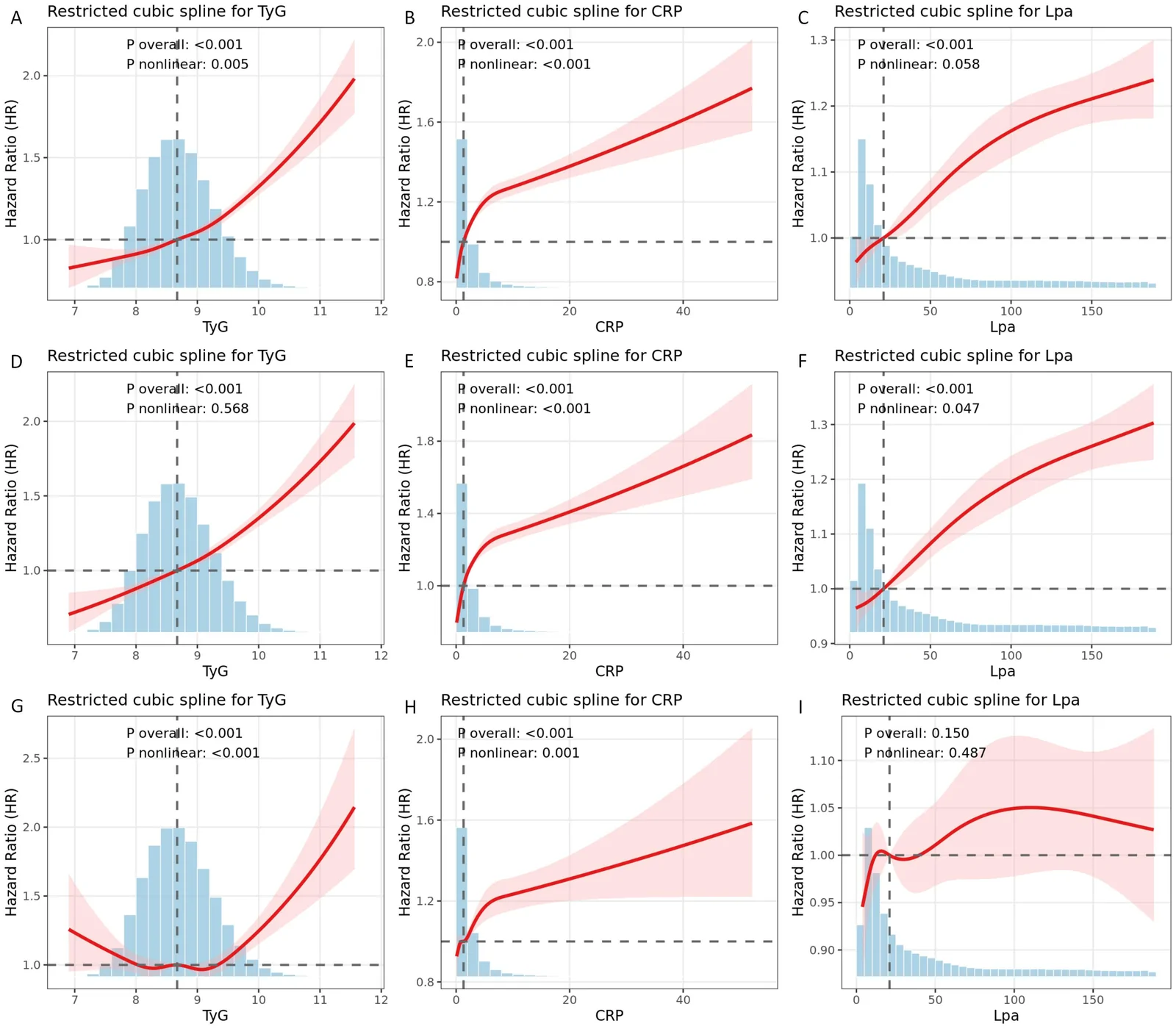
Dose response relationship of TyG, hs-CRP, and Lp(a) with atherosclerotic cardiovascular disease and its subtypes. **(A)** TyG and incident ASCVD; **(B)** hs-CRP and incident ASCVD; **(C)** Lp(a) and incident ASCVD; **(D)** TyG and incident CHD; **(E)** hs-CRP and incident CHD; **(F)** Lp(a) and incident CHD; Panel **(G)** TyG and incident stroke; **(H)** hs-CRP and incident stroke; **(I)** Lp(a) and incident stroke. Dose–response associations were evaluated using restricted cubic spline models. Models were adjusted for age, sex, Townsend Deprivation Index, ethnicity, current smoking status, physical activity, systolic blood pressure, body mass index, creatinine, antihypertensive medication use, lipid-lowering medication use, and the other two biomarkers, as applicable. CHD and stroke were analysed as exploratory subtype outcomes. ASCVD, atherosclerotic cardiovascular disease; CHD, coronary heart disease; TyG, triglyceride–glucose; hs-CRP, high-sensitivity C-reactive protein; Lp(a), lipoprotein(a).

**Figure 4** presents the multivariable-adjusted dose–response relationships between the three biomarkers and ASCVD and its subtypes, evaluated using RCS models. The TyG index showed significant nonlinear associations with ASCVD (*P* for nonlinear = 0.005) and stroke (*P* for nonlinear < 0.001), but not with CHD (*P* for nonlinear = 0.568). hs-CRP exhibited significant nonlinear dose–response relationships across all outcomes (all *P* for nonlinear < 0.05). Lp(a) showed an approximately linear association with ASCVD (*P* for nonlinear = 0.058), a nonlinear association with CHD (*P* for nonlinear = 0.047), and no significant overall association with stroke (*P* overall = 0.150).

### Joint associations of TyG, hs-CRP, and Lp(a) concentrations on ASCVD events and their subtypes

**Figure 5:**
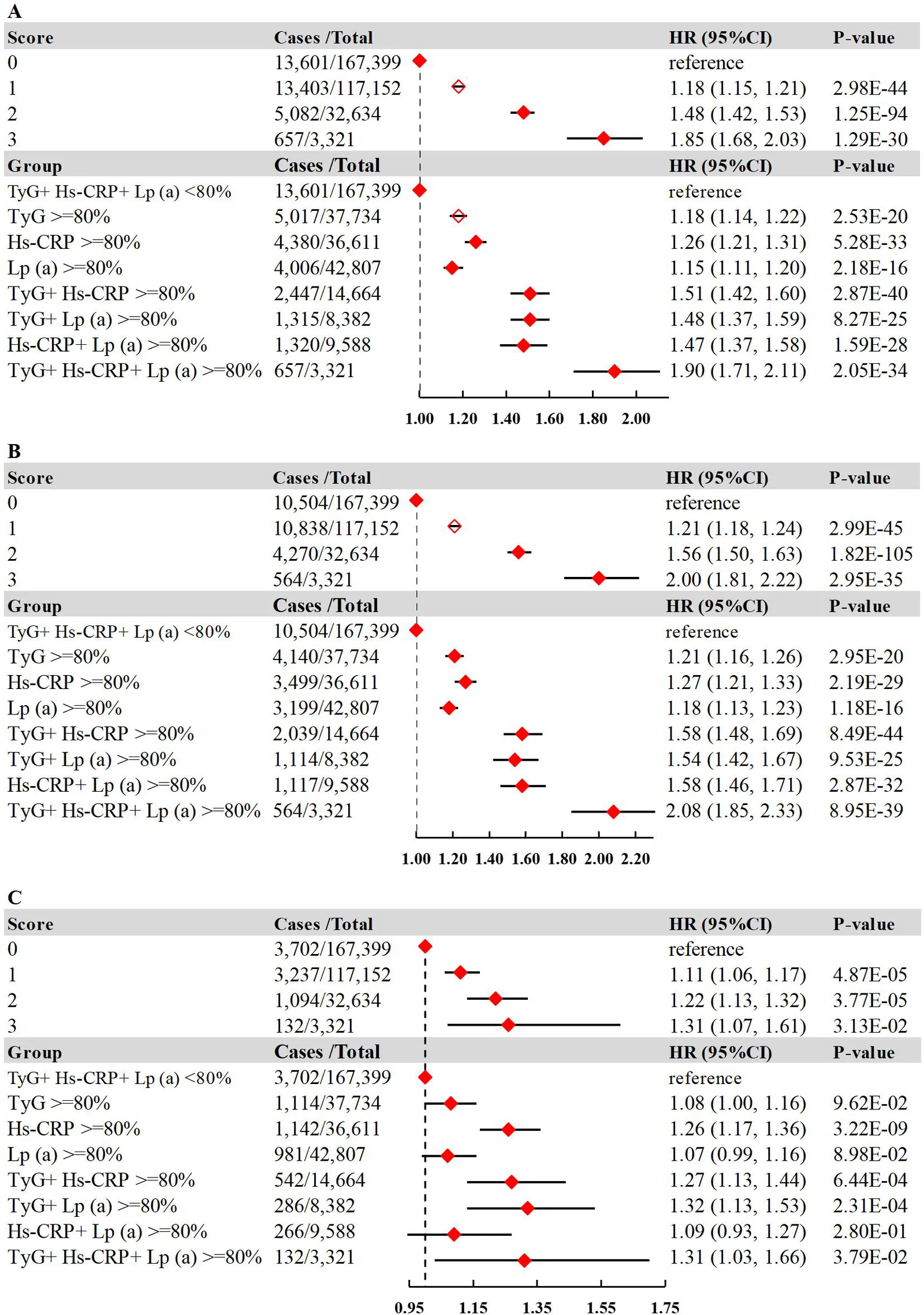
Joint associations of TyG, hs-CRP, and Lp(a) concentrations on ASCVD events and their subtypes. **(A)** Joint associations of TyG, hs-CRP, and Lp(a) concentrations on ASCVD events; **(B)** Joint associations of TyG, hs-CRP, and Lp(a) concentrations on CHD events; **(C)** Joint associations of TyG, hs-CRP, and Lp(a) concentrations on stroke events. Elevated biomarkers were defined as values in the fifth quintile of TyG, hs-CRP, or Lp(a). Model was adjusted for age, sex, TDI, ethnicity, current smoking status, physical activity, systolic blood pressure, BMI, creatinine, use of antihypertensive and lipid-lowering medications. TyG, triglyceride–glucose; hs-CRP, high-sensitivity C-reactive protein; Lp(a), lipoprotein(a); ASCVD, atherosclerotic cardiovascular disease; CHD, coronary heart disease; TDI, Townsend deprivation index; BMI, body mass index; HR, hazard ratio; CI, confidence interval.

Elevated TyG, hs-CRP, and Lp(a) levels were each associated with higher ASCVD risk, with the highest estimated risk observed among participants with concurrent elevation of all three biomarkers. After multivariable adjustment, compared with participants with none of the biomarkers in the fifth quintile, the hazard ratio (HR) for ASCVD was 1.18 (95% CI, 1.15–1.21) among those with one biomarker in the fifth quintile, increasing to 1.48 (95% CI, 1.42–1.53) and 1.85 (95% CI, 1.68–2.03) among those with two and three biomarkers in the fifth quintile, respectively. Exploratory analyses of ASCVD subtypes, including CHD and stroke, showed directionally similar patterns, although these findings should be interpreted as hypothesis-generating because no formal adjustment for multiple comparisons was performed (**Figure 5** and **Supplementary Table 5**).

Compared with the reference group in which TyG, hs-CRP, and Lp(a) were all below the fifth quintile, multivariable-adjusted analyses showed that the HR for ASCVD was 1.18 (95% CI, 1.14–1.22) among participants with TyG in the fifth quintile only, 1.26 (95% CI, 1.21–1.31) among those with hs-CRP in the fifth quintile only, and 1.15 (95% CI, 1.11–1.20) among those with Lp(a) in the fifth quintile only. In joint exposure analyses, the HRs for ASCVD were 1.51 (95% CI, 1.42–1.60) for participants with both TyG and hs-CRP in the fifth quintile, 1.48 (95% CI, 1.37–1.59) for those with TyG and Lp(a) in the fifth quintile, and 1.47 (95% CI, 1.37–1.58) for those with hs-CRP and Lp(a) in the fifth quintile. The highest risk of ASCVD was observed when all three biomarkers, TyG, hs-CRP, and Lp(a), were in the fifth quintile (HR, 1.90; 95% CI, 1.71–2.11). Exploratory analyses of ASCVD subtypes suggested that the observed associations were more evident for CHD than for stroke (**Figure 5** and **Supplementary Table 6**).

### Stratified analyses and sensitivity analyses

Stratified and sensitivity analyses were restricted to the primary ASCVD outcome. Exploratory subgroup analyses by sex and age group suggested potential heterogeneity in selected biomarker and ASCVD associations. However, these interaction findings should be regarded as hypothesis-generating because no formal adjustment for multiple comparisons was applied (**Supplementary Table 7**).

In sensitivity analyses, the overall patterns of association were consistent with those observed in the primary analyses, regardless of whether participants with a diagnosis of malignancy at or before baseline or those who experienced outcome events within the first two years of follow-up were excluded (**Supplementary Table 8**).

In incremental prediction analyses for 10-year incident ASCVD, the clinical model yielded a C-statistic of 0.712. Addition of TyG, hs-CRP, or Lp(a) individually resulted in small increases in discrimination, with C-statistics of 0.714, 0.715, and 0.713, respectively. The model including all three biomarkers showed the greatest improvement, with a C-statistic of 0.717 and a 10-year AUC of 0.735. Compared with the clinical model, the addition of all three biomarkers improved risk reclassification, with a continuous NRI of 18.65% and an IDI of 0.27%. The Brier score remained essentially unchanged, suggesting no material deterioration in overall prediction error (**Supplementary Table 9**).

## Discussion

In this large prospective cohort study with a median follow-up of 13.88 years, TyG and hs-CRP showed generally graded increases in ASCVD risk across quintiles, whereas the association for Lp(a) was more apparent in the higher quintiles. More importantly, irrespective of the specific distribution patterns of individual high-quintile biomarkers, ASCVD risk increased progressively with a greater number of high-risk biomarkers. When evaluated jointly, a greater burden of elevated biomarkers was associated with progressively higher ASCVD risk, with the highest estimated risk observed among participants with concurrent elevation of all three biomarkers. These findings suggest that metabolic, inflammatory, and Lp(a)-related biomarkers may provide partly non-redundant information for ASCVD risk stratification.

Consistent with previous studies, elevated levels of hs-CRP and Lp(a) have been associated with an increased risk of cardiovascular disease (11). Moreover, accumulating evidence suggests that the coexistence of inflammation and either modifiable or genetically determined lipoprotein burden is associated with a higher risk of cardiovascular events. A recent analysis from the Women’s Health Study reported that higher baseline levels of inflammatory and lipoprotein markers were associated with long-term risk of major adverse cardiovascular events (8), a finding that has been corroborated by subsequent studies. Similarly, metabolic dysfunction and insulin resistance, as reflected by the TyG index, have been associated with ASCVD risk in multiple populations (4, 24). However, to date, few studies have systematically evaluated the individual and joint contributions of insulin resistance, inflammation, and genetically determined lipoprotein burden within a single analytical framework. Building on existing evidence, our findings suggest that the TyG index may provide additional ASCVD risk information when evaluated alongside Lp(a) and hs-CRP. Exploratory subgroup analyses suggested potential heterogeneity by sex and age; however, because no formal adjustment for multiple comparisons was performed, these findings should be regarded as hypothesis-generating. Collectively, these results support the potential value of multi-biomarker profiling for ASCVD risk characterization and stratification, without implying mechanistic interaction among biomarker domains (4, 25).

Furthermore, the TyG index reflects a potentially modifiable component of risk, complementing Lp(a), which is largely genetically determined, and hs-CRP, which may be responsive to anti-inflammatory interventions. Overall, these observations suggest that ASCVD risk may be better characterized by considering multiple risk-related biomarker domains rather than any single biomarker alone. From a risk-stratification perspective, multi-biomarker profiling may help identify individuals with higher estimated ASCVD risk who might not be fully captured by conventional risk factors alone.

In addition, although traditional risk factors, such as age, blood pressure, dyslipidemia, and abnormalities in glucose metabolism, remain the cornerstone of ASCVD risk assessment, accumulating evidence indicates that ASCVD risk is not fully mitigated even among individuals with relatively well-controlled conventional risk factors (26). As a largely independent genetic risk factor characterized by lifelong exposure, Lp(a) may contribute to residual ASCVD risk even when metabolic and inflammatory pathways are well controlled (19, 27). Therefore, alongside systematic evaluation of traditional risk factors, incorporating genetic risk factors into a comprehensive risk assessment framework may facilitate a more complete characterization of residual risk across diverse populations.

In exploratory analyses of ASCVD subtypes, patterns of association differed across biomarkers and appeared generally more pronounced for CHD than for stroke, suggesting potential heterogeneity in biomarker–outcome associations across ASCVD manifestations. Overall, the associations of TyG, hs-CRP, and Lp(a) with CHD appeared stronger than those observed for stroke. This pattern is consistent with a more prominent contribution of insulin resistance, inflammatory activation, and lipoprotein-related mechanisms to the development and progression of coronary atherosclerosis (28–30). In contrast, the pathogenesis of stroke is more heterogeneous. In addition to atherosclerosis, stroke risk is influenced by multiple mechanisms, including small-vessel disease, blood pressure burden, and cardioembolic sources, which may partly attenuate the relative contribution of these biological pathways to overall stroke risk (31).

The TyG index is widely used as a surrogate marker of insulin resistance and captures a metabolic risk domain distinct from inflammatory and Lp(a)-related markers. By incorporating triglyceride and glucose concentrations, the TyG index reflects metabolic abnormalities related to insulin resistance that may not be fully captured by hs-CRP or Lp(a) (14). This may partly explain the additional risk information observed when these biomarkers were evaluated together. In the joint exposure analyses, a greater burden of elevated biomarkers was associated with progressively higher ASCVD risk, supporting the value of multi-biomarker risk characterization. However, these findings should not be interpreted as evidence of biological interaction, mechanistic synergy, or causal relationships among these biomarkers. Overall, the results support a risk-stratification framework incorporating metabolic, inflammatory, and Lp(a)-related biomarker domains (32, 33).

Finally, previous studies have shown that interventions targeting inflammatory pathways are associated with reduced cardiovascular event risk in selected populations (34, 35). In parallel, pharmacological therapies aimed at improving insulin resistance and metabolic status, including certain glucose-lowering and metabolic-modulating agents, have been associated with favorable cardiovascular outcomes (36, 37). In addition, several emerging therapies specifically targeting Lp(a) are currently under clinical investigation and have demonstrated substantial efficacy in lowering Lp(a) concentrations; however, their long-term cardiovascular benefits remain to be established in outcome-driven trials (38–40). In this context, the simultaneous assessment of TyG, hs-CRP, and Lp(a), representing distinct risk-related biomarker domains, may provide a more comprehensive characterization of ASCVD risk. Such an approach may help refine risk phenotyping and support more individualized ASCVD risk stratification.

## Strengths and limitations

The strengths of this study include its large sample size, long duration of follow-up, comprehensive adjustment for potential confounders, and the systematic evaluation of joint biomarker associations and exploratory subgroup patterns. Nevertheless, several limitations should be acknowledged. First, biomarkers were measured only at baseline, precluding assessment of temporal variability over time. Second, exclusion of participants with missing biomarker data may have introduced selection bias. Because glucose, triglyceride, hs-CRP, and Lp(a) measurements were required to calculate the TyG index and define joint biomarker profiles, analyses were restricted to participants with complete biomarker data. Accordingly, the findings may be most directly applicable to individuals with available measurements of these biomarkers, and validation in cohorts with systematic biomarker assessment is warranted. Third, fasting before blood sampling was not uniformly required in the UK Biobank. Therefore, TyG values were derived from triglyceride and glucose measurements obtained under non-standardized fasting conditions, which may have introduced measurement error and should be considered when interpreting TyG-related associations. Fourth, although extensive adjustment was performed for a wide range of potential confounders, residual confounding cannot be completely excluded. Fifth, outcome misclassification is possible because incident events were identified using linked health records and ICD-10 codes. Because ICD-10 codes I20-I25 capture coronary heart disease rather than myocardial infarction alone, CHD and stroke were analyzed as exploratory subtype outcomes, whereas primary inference focused on composite ASCVD. Additionally, participants in the UK Biobank are predominantly of European ancestry, which may limit the generalizability of these findings to other ethnic groups or populations. External validation in multi-ethnic and multicentre cohorts is warranted before these biomarker profiles are incorporated into routine clinical risk assessment. Finally, given the observational nature of the cohort design, causal inferences cannot be established.

## Conclusion

Our findings indicate that TyG, hs-CRP, and Lp(a), reflecting metabolic, inflammatory, and Lp(a)-related biomarker domains, respectively, are each associated with ASCVD risk. When these biomarkers were considered jointly, ASCVD risk increased progressively with a greater burden of elevated biomarkers, identifying individuals with higher estimated risk. Together, these results support the potential value of incorporating metabolic, inflammatory, and Lp(a)-related biomarkers into ASCVD risk characterization and stratification.

## Data Availability

The datasets presented in this study can be found in online repositories. The names of the repository/repositories and accession number(s) can be found below: The dataset supporting the conclusions of this article is available in the public UK Biobank Resource (www.ukbiobank.ac.uk/).
