# Supplementary material for: Metabolic, inflammatory, and lipoprotein(a)-related risk profiling for incident ASCVD: a multi-biomarker study
Source: Front Endocrinol (Lausanne). 2026 Aug 14;17:1901020. doi: 10.3389/fendo.2026.1901020 (PMC13521831; doi:10.3389/fendo.2026.1901020)
Supplement: Supplementary file 1 [file SupplementaryFile1.pdf]

Supplemental Material

Table S1. Proportional hazards diagnostic tests based on scaled Schoenfeld residuals

Table S2. Full baseline characteristics of the study participants according to increasing quintiles of TyG, hs-CRP, and Lp(a)

Table S3. Baseline characteristics of the study participants according to biomarker combination patterns

Table S4. Associations of TyG, hs-CRP, and Lp(a) concentrations with incident ASCVD and their subtypes

Table S5. Associations of biomarker combination score defined by TyG, hs-CRP, and Lp(a) with incident ASCVD and its subtypes

Table S6. Associations of biomarker combination patterns defined by TyG, hs-CRP, and Lp(a) with incident ASCVD and its subtypes

Table S7. Stratified analyses of individual and joint biomarker associations with incident ASCVD by sex and age group

Table S8. Sensitivity analysisof individual and joint biomarker associations with incident ASCVD

Table S9. Incremental predictive performance of TyG, hs-CRP, and Lp(a), individually and jointly, for 10-year incident ASCVD

Figure S1. Scaled Schoenfeld residual plots for exposure terms in the Cox models

Table S1. Proportional hazards diagnostic tests based on scaled Schoenfeld residuals

Panel A. Group of TyG concentration

Variable	Chi square	P value	PH assumption
Group 1	-	-	-
Group 2	2.08294128	1.489529e-01	Satisfied
Group 3	0.18398801	6.679682e-01	Satisfied
Group 4	4.02351682	4.487006e-02	Violated
Group 5	25.71694659	3.953402e-07	Violated
Age	15.56570527	7.968693e-05	Violated
Sex	26.03661809	3.350028e-07	Violated
Ethnicity	1.88049572	1.702778e-01	Satisfied
TDI	0.87819060	3.486977e-01	Satisfied
Smoking status	1.86164652	1.724349e-01	Satisfied
Physical activities	0.01432387	9.047348e-01	Satisfied
SBP	28.57756486	9.001916e-08	Violated
BMI	5.31128094	2.118777e-02	Violated
Cr	17.49337181	2.883111e-05	Violated
hs-CRP	5.35321660	2.068408e-02	Violated
Lp(a)	0.00903876	9.242573e-01	Satisfied
Antihypertension medicine	9.82697299	1.719711e-03	Violated
Lipid-lowering medicine	15.39439009	8.724693e-05	Violated
Global	116.06015839	6.020654e-16	Violated

Panel B. Group of hs-CRP concentration

Variable	Chi square	P value	PH assumption
Group 1	-	-	-
Group 2	5.114804e+00	2.372254e-02	Violated

Group 3	1.713466e-01	6.789178e-01	Satisfied
Group 4	3.428518e+00	6.407958e-02	Satisfied
Group 5	1.514749e+01	9.943022e-05	Violated
Age	1.567667e+01	7.514543e-05	Violated
Sex	2.561434e+01	4.169288e-07	Violated
Ethnicity	1.944930e+00	1.631343e-01	Satisfied
TDI	9.388499e-01	3.325738e-01	Satisfied
Smoking status	1.877976e+00	1.705644e-01	Satisfied
Physical activities	2.069890e-02	8.856023e-01	Satisfied
SBP	2.880478e+01	8.005338e-08	Violated
BMI	6.089264e+00	1.360058e-02	Violated
Cr	1.893179e+01	1.354762e-05	Violated
TyG	2.636629e+01	2.824267e-07	Violated
Lp(a)	9.988515e-03	9.203899e-01	Satisfied
Antihypertension medicine	9.977243e+00	1.584868e-03	Violated
Lipid-lowering medicine	1.556213e+01	7.983790e-05	Violated
Global	1.280833e+02	3.358007e-18	Violated

Panel C. Group of Lp(a) concentration

Variable	Chi square	P value	PH assumption
Group 1	-	-	-
Group 2	0.04451514	8.328981e-01	Satisfied
Group 3	0.03040137	8.615826e-01	Satisfied
Group 4	3.88200252	4.880631e-02	Violated
Group 5	0.64432367	4.221495e-01	Satisfied
Age	15.73325754	7.293056e-05	Violated
Sex	26.12348164	3.202654e-07	Violated

Ethnicity	1.88271300	1.700261e-01	Satisfied
TDI	0.86763836	3.516099e-01	Satisfied
Smoking status	1.85562325	1.731308e-01	Satisfied
Physical activities	0.01373816	9.066937e-01	Satisfied
SBP	28.73758308	8.287961e-08	Violated
BMI	5.29756006	2.135532e-02	Violated
Cr	16.91787218	3.903235e-05	Violated
hs-CRP	5.42283618	1.987505e-02	Violated
TyG	26.08909510	3.260201e-07	Violated
Antihypertension medicine	9.90359168	1.649565e-03	Violated
Lipid-lowering medicine	15.36143854	8.878180e-05	Violated
Global	114.33671737	1.258145e-15	Violated

Panel D. Group of biomarker combination patterns defined by TyG, hs-CRP, and Lp(a)

Variable	Chi square	P value	PH assumption
Group 1	-	-	-
Group 2	18.50718180	1.692653e-05	Violated
Group 3	2.57247384	1.087376e-01	Satisfied
Group 4	10.88510745	9.694056e-04	Violated
Group 5	3.06502063	7.999415e-02	Satisfied
Group 6	0.09240104	7.611466e-01	Satisfied
Group 7	6.12866263	1.330074e-02	Violated
Group 8	1.83558152	1.754697e-01	Satisfied
Age	15.30076050	9.167959e-05	Violated
Sex	25.42505871	4.599052e-07	Violated
Ethnicity	1.88220105	1.700842e-01	Satisfied
TDI	0.86412045	3.525882e-01	Satisfied

Smoking status	1.91602233	1.662958e-01	Satisfied
Physical activities	0.01556235	9.007221e-01	Satisfied
SBP	27.92667535	1.260007e-07	Violated
BMI	5.14058128	2.337282e-02	Violated
Cr	17.37561510	3.067362e-05	Violated
Antihypertension medicine	9.72569239	1.817099e-03	Violated
Lipid-lowering medicine	15.39756418	8.710050e-05	Violated
Global	127.32790231	1.234000e-17	Violated

Abbreviations: TyG, triglyceride–glucose; hs-CRP, high-sensitivity C-reactive protein; Lp(a), lipoprotein(a); TDI, Townsend Deprivation Index; SBP, systolic blood pressure; BMI, body mass index; Cr, creatinine.

Table S2. Full baseline characteristics of the study participants according to increasing quintiles of TyG, hs-CRP, and Lp(a)

Panel A. According to increasing quintiles of TyG

	Total (N = 320,506)	Quintile 1 (N = 64,101)	Quintile 2 (N = 64,101)	Quintile 3 (N = 64,102)	Quintile 4 (N = 64,101)	Quintile 5 (N = 64,101)	*P* value
Age, y	56.17±8.11	53.50±8.23	55.98±8.09	56.92±7.94	57.34±7.83	57.10±7.80	<.001
Men, N (%)	143,006 (44.62)	19,552 (30.50)	24,227 (37.80)	28,239 (44.05)	32,359 (50.48)	38,629 (60.26)	<.001
Ethnicity							<.001
White, N (%)	301,171 (93.97)	59,153 (92.28)	60,310 (94.09)	60,630 (94.58)	60,698 (94.69)	60,380 (94.20)	
Non-White, N (%)	19,335 (6.03)	4,948 (7.72)	3,791 (5.91)	3,472 (5.42)	3,403 (5.31)	3,721 (5.80)	
TDI	-1.35±3.06	-1.34±3.09	-1.43±3.02	-1.41±3.02	-1.39±3.05	-1.19±3.12	<.001
Current smokers, N (%)	33,353 (10.41)	5,410 (8.44)	5,979 (9.33)	6,490 (10.12)	7,050 (11.00)	8,424 (13.14)	<.001
Physical activities							<.001
Low, N (%)	95,410 (29.77)	16,041 (25.02)	17,741 (27.68)	19,064 (29.74)	20,294 (31.66)	22,270 (34.74)	
Moderate, N (%)	151,658 (47.32)	30,973 (48.32)	30,881 (48.18)	30,458 (47.51)	30,008 (46.81)	29,338 (45.77)	
High, N (%)	73,438 (22.91)	17,087 (26.66)	15,479 (24.15)	14,580 (22.75)	13,799 (21.53)	12,493 (19.49)	
TyG	8.70±0.56	7.96±0.20	8.38±0.09	8.67±0.08	8.98±0.10	9.52±0.32	<.001
hs-CRP, mg/L	1.31 (0.65–2.73)	0.81 (0.42–1.72)	1.10 (0.56–2.29)	1.35 (0.69–2.78)	1.59 (0.82–3.17)	1.88 (0.99–3.60)	
Lpa, nmol/L	20.99 (9.55–61.50)	22.69 (9.89–69.82)	22.16 (9.95–63.96)	21.47 (9.81–59.82)	20.95 (9.60–59.25)	18.10 (8.64–55.20)	
SBP, mmHg	137.66±18.60	131.34±18.32	135.93±18.47	138.37±18.37	140.31±18.08	142.34±17.80	<.001
DBP, mmHg	82.36±10.10	79.11±9.91	81.35±9.90	82.68±9.91	83.81±9.89	84.88±9.88	<.001
BMI, kg/m2	27.26±4.72	24.87±3.90	26.26±4.36	27.37±4.56	28.32±4.64	29.49±4.70	<.001
HbA1c, mmol/mol	35.81±6.28	34.05±4.39	34.80±4.40	35.37±4.67	36.12±5.29	38.69±9.80	<.001

Glu, mmol/l	5.08±1.12	4.69±0.55	4.89±0.57	4.99±0.66	5.11±0.85	5.74±1.96	<.001
TC, mmol/l	5.76±1.10	5.34±0.96	5.63±1.00	5.78±1.05	5.94±1.11	6.09±1.23	<.001
LDL-C, mmol/l	3.61±0.84	3.20±0.70	3.50±0.75	3.67±0.80	3.81±0.85	3.86±0.92	<.001
HDL-C, mmol/l	1.46±0.38	1.70±0.39	1.57±0.37	1.46±0.34	1.35±0.31	1.21±0.27	<.001
TG, mmol/l	1.73±1.00	0.79±0.16	1.13±0.16	1.49±0.21	1.99±0.32	3.24±1.08	<.001
Creatinine, umol/L	71.77±17.16	68.51±14.58	70.47±16.25	71.86±15.28	73.35±18.33	74.68±20.06	<.001
Anti-hypertensive drugs, N (%)	32,926 (10.27)	3,950 (6.16)	5,747 (8.97)	6,889 (10.75)	7,889 (12.31)	8,451 (13.18)	<.001
Lipid-lowering drugs, N (%)	40,395 (12.60)	4,223 (6.59)	6,057 (9.45)	7,641 (11.92)	9,327 (14.55)	13,147 (20.51)	<.001

Panel B. According to increasing quintiles of hs-CRP

	Total (N = 320,506)	Quintile 1 (N = 63,626)	Quintile 2 (N = 64,746)	Quintile 3 (N = 63,871)	Quintile 4 (N = 64,079)	Quintile 5 (N = 64,184)	*P* value
Age, y	56.17±8.11	54.25±8.12	55.81±8.08	56.63±8.05	57.09±7.95	57.05±7.98	<.001
Men, N (%)	143,006 (44.62)	27,280 (42.88)	30,894 (47.72)	30,499 (47.75)	28,972 (45.21)	25,361 (39.51)	<.001
Ethnicity							<.001
White, N (%)	301,171 (93.97)	59,750 (93.91)	61,048 (94.29)	60,142 (94.16)	60,249 (94.02)	59,982 (93.45)	<.001
Non-White, N (%)	19,335 (6.03)	3,876 (6.09)	3,698 (5.71)	3,729 (5.84)	3,830 (5.98)	4,202 (6.55)	<.001
TDI	-1.35±3.06	-1.54±2.96	-1.55±2.96	-1.46±3.01	-1.29±3.09	-0.93±3.24	<.001
Current smokers, N (%)	33,353 (10.41)	4,903 (7.71)	5,562 (8.59)	6,221 (9.74)	7,375 (11.51)	9,292 (14.48)	<.001
Physical activities							<.001
Low, N (%)	95,410 (29.77)	14,797 (23.26)	17,076 (26.37)	18,400 (28.81)	20,881 (32.59)	24,256 (37.79)	

Moderate, N (%)	151,658 (47.32)	31,378 (49.32)	31,347 (48.42)	30,515 (47.78)	29,689 (46.33)	28,729 (44.76)	
High, N (%)	73,438 (22.91)	17,451 (27.43)	16,323 (25.21)	14,956 (23.42)	13,509 (21.08)	11,199 (17.45)	
TyG	8.70±0.56	8.45±0.51	8.63±0.54	8.73±0.54	8.82±0.55	8.87±0.56	<.001
hs-CRP, mg/L	1.31 (0.65–2.73)	0.36 (0.26–0.45)	0.75 (0.65–0.87)	1.31 (1.15–1.50)	2.31 (1.99–2.73)	5.46 (4.10–8.68)	<.001
Lpa, nmol/L	20.99 (9.55–61.50)	19.88 (9.27–61.02)	20.30 (9.41–60.79)	21.00 (9.60–60.70)	21.30 (9.60–61.81)	22.50 (9.90–63.10)	<.001
SBP, mmHg	137.66±18.60	132.74±18.10	136.39±18.18	138.50±18.51	139.95±18.51	140.69±18.59	<.001
DBP, mmHg	82.36±10.10	79.48±9.82	81.56±9.89	82.72±9.96	83.67±10.00	84.38±10.08	<.001
BMI, kg/m2	27.26±4.72	24.32±3.22	26.01±3.53	27.21±3.91	28.47±4.39	30.29±5.76	<.001
HbA1c, mmol/mol	35.81±6.28	34.43±4.98	35.07±5.35	35.67±5.83	36.32±6.45	37.53±7.90	<.001
Glu, mmol/l	5.08±1.12	4.96±0.88	5.01±0.94	5.07±1.05	5.13±1.16	5.25±1.44	<.001
TC, mmol/l	5.76±1.10	5.62±1.04	5.74±1.08	5.81±1.10	5.85±1.13	5.76±1.15	<.001
LDL-C, mmol/l	3.61±0.84	3.45±0.79	3.58±0.82	3.65±0.84	3.70±0.86	3.66±0.87	<.001
HDL-C, mmol/l	1.46±0.38	1.58±0.39	1.50±0.38	1.45±0.37	1.41±0.36	1.36±0.35	<.001
TG, mmol/l	1.73±1.00	1.35±0.77	1.62±0.93	1.78±1.01	1.92±1.06	1.97±1.07	<.001
Creatinine, umol/L	71.77±17.16	70.37±14.37	72.07±15.04	72.57±16.09	72.29±17.11	71.57±22.00	<.001
Anti-hypertensive drugs, N (%)	32,926 (10.27)	3,465 (5.45)	4,952 (7.65)	6,196 (9.70)	7,936 (12.38)	10,377 (16.17)	<.001
Lipid-lowering drugs, N (%)	40,395 (12.60)	6,165 (9.69)	8,094 (12.50)	8,538 (13.37)	8,800 (13.73)	8,798 (13.71)	<.001

Panel C. According to increasing quintiles of Lp(a)

	Total (N = 320,506)	Quintile 1 (N = 64,104)	Quintile 2 (N = 64,108)	Quintile 3 (N = 64,092)	Quintile 4 (N = 64,104)	Quintile 5 (N = 64,098)	*P* value
Age, y	56.17±8.11	55.88±8.19	55.97±8.13	56.52±8.02	56.49±8.03	55.98±8.13	<.001

Men, N (%)	143,006 (44.62)	30,386 (47.40)	29,803 (46.49)	27,856 (43.46)	26,463 (41.28)	28,498 (44.46)	<.001
Ethnicity							<.001
White, N (%)	301,171 (93.97)	61,958 (96.65)	61,467 (95.88)	60,585 (94.53)	57,713 (90.03)	59,448 (92.75)	<.001
Non-White, N (%)	19,335 (6.03)	2,146 (3.35)	2,641 (4.12)	3,507 (5.47)	6,391 (9.97)	4,650 (7.25)	<.001
TDI	-1.35±3.06	-1.41±3.01	-1.43±3.01	-1.41±3.02	-1.20±3.16	-1.31±3.10	<.001
Current smokers, N (%)	33,353 (10.41)	6,710 (10.47)	6,681 (10.42)	6,573 (10.26)	6,726 (10.49)	6,663 (10.40)	0.002
Physical activities							<.001
Low, N (%)	95,410 (29.77)	19,136 (29.85)	18,883 (29.45)	19,013 (29.67)	19,452 (30.34)	18,926 (29.53)	<.001
Moderate, N (%)	151,658 (47.32)	30,184 (47.09)	30,394 (47.41)	30,428 (47.48)	30,438 (47.48)	30,214 (47.14)	<.001
High, N (%)	73,438 (22.91)	14,784 (23.06)	14,831 (23.13)	14,651 (22.86)	14,214 (22.17)	14,958 (23.34)	<.001
TyG	8.70±0.56	8.75±0.59	8.72±0.57	8.70±0.54	8.68±0.54	8.67±0.56	<.001
hs-CRP, mg/L	1.31 (0.65–2.73)	1.28 (0.63–2.63)	1.27 (0.63–2.65)	1.32 (0.65–2.75)	1.38 (0.68–2.89)	1.31 (0.65–2.74)	
Lpa, nmol/L	20.99 (9.55–61.50)	5.70 (4.67–6.90)	11.08 (9.55–12.75)	20.99 (17.50–25.70)	48.90 (39.54–61.50)	130.59 (105.65–157.90)	
SBP, mmHg	137.66±18.60	137.76±18.62	137.49±18.51	137.68±18.60	137.68±18.67	137.68±18.60	0.115
DBP, mmHg	82.36±10.10	82.41±10.11	82.31±10.13	82.29±10.03	82.38±10.10	82.44±10.13	0.034
BMI, kg/m2	27.26±4.72	27.28±4.77	27.21±4.70	27.18±4.62	27.33±4.74	27.30±4.76	<.001
HbA1c, mmol/mol	35.81±6.28	35.85±6.65	35.68±6.39	35.73±5.92	35.94±6.18	35.83±6.23	<.001
Glu, mmol/l	5.08±1.12	5.13±1.23	5.09±1.12	5.06±1.05	5.06±1.05	5.08±1.11	<.001
TC, mmol/l	5.76±1.10	5.63±1.10	5.68±1.08	5.79±1.09	5.84±1.13	5.83±1.10	<.001
LDL-C, mmol/l	3.61±0.84	3.49±0.84	3.54±0.82	3.64±0.83	3.68±0.86	3.68±0.83	<.001
HDL-C, mmol/l	1.46±0.38	1.45±0.39	1.45±0.38	1.46±0.38	1.46±0.37	1.47±0.38	<.001

TG, mmol/l	1.73±1.00	1.81±1.08	1.76±1.02	1.72±0.97	1.69±0.95	1.67±0.97	<.001
Creatinine, umol/L	71.77±17.16	71.70±15.93	71.88±16.69	71.62±16.04	71.66±19.03	72.01±17.89	<.001
Anti-hypertensive drugs, N (%)	32,926 (10.27)	6,590 (10.28)	6,611 (10.31)	6,513 (10.16)	6,746 (10.52)	6,466 (10.09)	0.102
Lipid-lowering drugs, N (%)	40,395 (12.60)	8,149 (12.71)	7,565 (11.80)	7,870 (12.28)	8,502 (13.26)	8,309 (12.96)	<.001

Abbreviations: TyG, triglyceride–glucose; hs-CRP, high-sensitivity C-reactive protein; Lp(a), lipoprotein(a); TDI, Townsend Deprivation Index; SBP, systolic blood pressure; DBP, diastolic blood pressure; BMI, body mass index; HbA1c, glycated hemoglobin; Glu, glucose; TC, total cholesterol; LDL-C, low-density lipoprotein cholesterol; HDL-C, high-density lipoprotein cholesterol; TG, triglyceride.

Table S3. Baseline characteristics of the study participants according to biomarker combination patterns

	Biomarker combination patterns									
	Total (N = 320,506)	Group 1 (N = 167,399)	Group 2 (N = 37,734)	Group 3 (N = 36,611)	Group 4 (N = 42,807)	Group 5 (N = 14,664)	Group 6 (N = 8,382)	Group 7 (N = 9,588)	Group 8 (N = 3,321)	*P* value
Age, y	56.17±8.11	55.76±8.16	57.02±7.84	56.95±8.08	55.58±8.17	57.49±7.63	56.66±7.88	56.68±8.14	57.35±7.81	<.001
Men, N (%)	143,006 (44.62)	69,405 (41.46)	24,684 (65.42)	13,523 (36.94)	18,044 (42.15)	6,896 (47.03)	5,512 (65.76)	3,405 (35.51)	1,537 (46.28)	<.001
Ethnicity										<.001
White, N (%)	301,171 (93.97)	158,014 (94.39)	35,536 (94.18)	34,377 (93.90)	39,707 (92.76)	13,796 (94.08)	7,932 (94.63)	8,693 (90.67)	3,116 (93.83)	
Non-White, N (%)	19,335 (6.03)	9,385 (5.61)	2,198 (5.82)	2,234 (6.10)	3,100 (7.24)	868 (5.92)	450 (5.37)	895 (9.33)	205 (6.17)	<.001
TDI	-1.35±3.06	-1.50±2.98	-1.35±3.05	-1.01±3.22	-1.42±3.05	-0.76±3.26	-1.39±3.01	-0.93±3.28	-0.80±3.30	<.001
Current smokers, N (%)	33,353 (10.41)	14,817 (8.85)	4,415 (11.70)	4,968 (13.57)	3,864 (9.03)	2,490 (16.98)	965 (11.51)	1,280 (13.35)	554 (16.68)	<.001
Physical activities										<.001
Low, N (%)	95,410 (29.77)	44,942 (26.85)	12,178 (32.27)	13,270 (36.25)	11,385 (26.60)	6,094 (41.56)	2,649 (31.60)	3,543 (36.95)	1,349 (40.62)	<.001
Moderate, N (%)	151,658 (47.32)	80,778 (48.25)	17,658 (46.80)	16,679 (45.56)	20,620 (48.17)	6,329 (43.16)	3,873 (46.21)	4,243 (44.25)	1,478 (44.50)	<.001
High, N (%)	73,438	41,679	7,898	6,662	10,802 (25.23)	2,241	1,860 (22.19)	1,802 (18.79)	494 (14.88)	<.00

	(22.91)	(24.90)	(20.93)	(18.20)		(15.28)				1
TyG	8.70±0.56	8.48±0.40	9.51±0.30	8.61±0.36	8.45±0.40	9.55±0.35	9.50±0.30	8.58±0.37	9.55±0.35	<.001
hs-CRP, mg/L	1.31 (0.65–2.73)	0.93 (0.51–1.63)	1.33 (0.80–2.07)	5.49 (4.11–8.88)	0.94 (0.51–1.64)	5.40 (4.10–8.28)	1.33 (0.80–2.07)	5.48 (4.11–8.71)	5.36 (4.04–8.34)	<.001
Lpa, nmol/L	20.99 (9.55–61.50)	15.00 (8.29–32.00)	13.30 (7.50–28.10)	16.44 (8.70–35.00)	130.30 (105.70–157.50)	14.10 (7.80–30.50)	129.90 (104.84–156.80)	132.91 (106.52–160.10)	129.67 (104.80–157.90)	<.001
SBP, mmHg	137.66±18.60	135.77±18.55	141.88±17.62	139.62±18.61	135.91±18.48	143.36±18.19	142.09±17.68	139.66±18.78	143.58±18.12	<.001
DBP, mmHg	82.36±10.10	81.23±9.98	84.53±9.81	83.86±10.05	81.38±10.04	85.64±10.03	84.79±9.78	83.97±10.05	85.62±10.07	<.001
BMI, kg/m2	27.26±4.72	26.02±3.94	28.63±4.03	29.71±5.73	26.10±3.98	31.64±5.52	28.69±4.04	29.90±5.85	31.78±5.49	<.001
HbA1c, mmol/mol	35.81±6.28	34.81±4.59	37.84±8.82	36.24±5.30	34.92±4.64	40.81±11.56	37.90±8.89	36.30±5.25	40.88±12.01	<.001
Glu, mmol/l	5.08±1.12	4.91±0.67	5.64±1.78	4.95±0.70	4.91±0.69	6.00±2.29	5.65±1.84	4.97±0.72	6.06±2.38	<.001
TC, mmol/l	5.76±1.10	5.66±1.05	6.06±1.22	5.60±1.07	5.77±1.05	6.09±1.26	6.18±1.21	5.71±1.08	6.19±1.23	<.001
LDL-C, mmol/l	3.61±0.84	3.52±0.81	3.83±0.91	3.54±0.83	3.61±0.80	3.88±0.94	3.93±0.89	3.64±0.83	3.97±0.91	<.001
HDL-C, mmol/l	1.46±0.38	1.54±0.38	1.21±0.27	1.42±0.36	1.55±0.38	1.19±0.27	1.22±0.26	1.42±0.36	1.19±0.26	<.001
TG, mmol/l	1.73±1.00	1.33±0.50	3.24±1.07	1.48±0.49	1.30±0.49	3.24±1.10	3.22±1.06	1.44±0.49	3.21±1.09	<.001
Creatinine, umol/L	71.77±17.1	70.94±15.1	75.37±17.6	71.05±19.6	71.41±14.97	72.85±24.7	75.66±18.22	71.27±24.49	72.58±26.06	<.00

	6	6	1	0		2				1
Anti-hypertensive drugs, N (%)	32,926 (10.27)	13,889 (8.30)	4,292 (11.37)	5,610 (15.32)	3,469 (8.10)	2,669 (18.20)	899 (10.73)	1,507 (15.72)	591 (17.80)	<.001
Lipid-lowering drugs, N (%)	40,395 (12.60)	17,252 (10.31)	7,836 (20.77)	4,105 (11.21)	4,737 (11.07)	2,893 (19.73)	1,772 (21.14)	1,154 (12.04)	646 (19.45)	<.001

Biomarker combination patterns: (1) TyG, hs-CRP, and Lp(a) all below the fifth quintile; (2) TyG in the fifth quintile only; (3) hs-CRP in the fifth quintile only; (4) Lp(a) in the fifth quintile only; (5) TyG and hs-CRP in the fifth quintile; (6) TyG and Lp(a) in the fifth quintile; (7) hs-CRP and Lp(a) in the fifth quintile; and (8) TyG, hs-CRP, and Lp(a) all in the fifth quintile.

Abbreviations: TyG, triglyceride–glucose; hs-CRP, high-sensitivity C-reactive protein; Lp(a), lipoprotein(a); TDI, Townsend deprivation index; SBP, systolic blood pressure; DBP, diastolic blood pressure; BMI, body mass index; HbA1c, glycated hemoglobin; Glu, glucose; TC, total cholesterol; LDL-C, low-density lipoprotein cholesterol; HDL-C, high-density lipoprotein cholesterol; TG, triglyceride.

Table S4. Associations of TyG, hs-CRP, and Lp(a) concentrations with incident ASCVD events and their subtypes

Panel A. ASCVD

	Quintile 1	Quintile 2	Quintile 3	Quintile 4	Quintile 5
TyG concentrations					
Cases/ Total	3,984/64,101	5,317/64,101	6,515/64,102	7,491/64,101	9,436/64,101
IR, per 1,000 person-years	4.59	6.24	7.74	8.97	11.50
Model 1, HR (95%CI)	1 (reference)	1.05 (1.00, 1.10)	1.08 (1.03, 1.13)	1.13 (1.08, 1.18)	1.33 (1.27, 1.39)
Model 2, HR (95%CI)	1 (reference)	1.02 (0.97, 1.07)	1.06 (1.01, 1.11)	1.10 (1.05, 1.15)	1.27 (1.21, 1.33)
Model 3, HR (95%CI)	1 (reference)	1.03 (0.98, 1.08)	1.08 (1.04, 1.13)	1.13 (1.08, 1.18)	1.32 (1.27, 1.38)
hs-CRP concentrations					
Cases/ Total	4,155/63,626	5,655/64,746	6,597/63,871	7,532/64,079	8,804/64,184
IR, per 1,000 person-years	4.83	6.55	7.83	9.03	10.80
Model 1, HR (95%CI)	1 (reference)	1.07 (1.02, 1.13)	1.14 (1.09, 1.19)	1.23 (1.17, 1.29)	1.38 (1.32, 1.45)
Model 2, HR (95%CI)	1 (reference)	1.05 (0.99, 1.10)	1.11 (1.05, 1.16)	1.19 (1.14, 1.25)	1.36 (1.29, 1.43)
Model 3, HR (95%CI)	1 (reference)	1.04 (0.99, 1.10)	1.10 (1.05, 1.16)	1.19 (1.14, 1.25)	1.36 (1.30, 1.43)
Lp(a) concentrations					
Cases/ Total	6,214/64,104	6,253/64,108	6,243/64,092	6,735/64,104	7,298/64,098
IR, per 1,000 person-years	7.35	7.39	7.39	8.01	8.69
Model 1, HR (95%CI)	1 (reference)	1.02 (0.99, 1.06)	1.01 (0.97, 1.04)	1.09 (1.06, 1.13)	1.22 (1.18, 1.26)
Model 2, HR (95%CI)	1 (reference)	1.02 (0.99, 1.06)	1.01 (0.98, 1.05)	1.10 (1.07, 1.14)	1.23 (1.19, 1.27)
Model 3, HR (95%CI)	1 (reference)	1.02 (0.99, 1.06)	1.02 (0.98, 1.05)	1.11 (1.07, 1.15)	1.23 (1.19, 1.27)

Panel B. CHD

	Quintile 1	Quintile 2	Quintile 3	Quintile 4	Quintile 5
TyG concentrations					
Cases/ Total	2,974/64,101	4,119/64,101	5,137/64,102	6,089/64,101	7,857/64,101
IR, per 1,000 person-years	3.41	4.80	6.05	7.24	9.51

Model 1, HR (95%CI)	1 (reference)	1.06 (1.01, 1.12)	1.11 (1.06, 1.17)	1.18 (1.12, 1.24)	1.41 (1.35, 1.49)
Model 2, HR (95%CI)	1 (reference)	1.04 (0.98, 1.09)	1.10 (1.05, 1.15)	1.17 (1.12, 1.23)	1.39 (1.32, 1.46)
Model 3, HR (95%CI)	1 (reference)	1.04 (0.99, 1.10)	1.11 (1.06, 1.17)	1.19 (1.13, 1.25)	1.41 (1.34, 1.48)
hs-CRP concentrations					
Cases/ Total	3,190/63,626	4,395/64,746	5,281/63,871	6,091/64,079	7,219/64,184
IR, per 1,000 person-years	3.69	5.06	6.22	7.24	8.75
Model 1, HR (95%CI)	1 (reference)	1.05 (1.00, 1.11)	1.15 (1.09, 1.21)	1.24 (1.18, 1.31)	1.40 (1.32, 1.47)
Model 2, HR (95%CI)	1 (reference)	1.02 (0.97, 1.08)	1.11 (1.05, 1.17)	1.20 (1.14, 1.27)	1.37 (1.30, 1.45)
Model 3, HR (95%CI)	1 (reference)	1.02 (0.97, 1.08)	1.11 (1.05, 1.17)	1.20 (1.14, 1.27)	1.38 (1.31, 1.46)
Lp(a) concentrations					
Cases/ Total	4,920/64,104	4,940/64,108	4,920/64,092	5,402/64,104	5,994/64,098
IR, per 1,000 person-years	5.78	5.80	5.78	6.38	7.08
Model 1, HR (95%CI)	1 (reference)	1.02 (0.98, 1.06)	1.01 (0.97, 1.05)	1.12 (1.07, 1.16)	1.26 (1.22, 1.31)
Model 2, HR (95%CI)	1 (reference)	1.02 (0.98, 1.07)	1.02 (0.98, 1.06)	1.13 (1.08, 1.17)	1.28 (1.23, 1.33)
Model 3, HR (95%CI)	1 (reference)	1.02 (0.98, 1.07)	1.02 (0.98, 1.06)	1.13 (1.09, 1.18)	1.28 (1.23, 1.33)

Panel C. Stroke

	Quintile 1	Quintile 2	Quintile 3	Quintile 4	Quintile 5
TyG concentrations					
Cases/ Total	1,186/64,101	1,470/64,101	1,697/64,102	1,738/64,101	2,074/64,101
IR, per 1,000 person-years	1.34	1.68	1.95	2.00	2.40
Model 1, HR (95%CI)	1 (reference)	1.03 (0.94, 1.13)	1.00 (0.92, 1.09)	0.95 (0.87, 1.03)	1.07 (0.99, 1.17)
Model 2, HR (95%CI)	1 (reference)	1.02 (0.94, 1.13)	1.00 (0.92, 1.09)	0.95 (0.87, 1.03)	1.07 (0.98, 1.17)
Model 3, HR (95%CI)	1 (reference)	1.03 (0.94, 1.12)	1.01 (0.92, 1.10)	0.95 (0.88, 1.04)	1.07 (0.99, 1.17)
hs-CRP concentrations					
	Quintile 1	Quintile 2	Quintile 3	Quintile 4	Quintile 5
Cases/ Total	1,146/63,626	1,504/64,746	1,613/63,871	1,820/64,079	2,082/64,184

IR, per 1,000 person-years	1.31	1.69	1.85	2.10	2.43
Model 1, HR (95%CI)	1 (reference)	1.10 (1.00, 1.21)	1.08 (0.99, 1.18)	1.17 (1.06, 1.28)	1.33 (1.21, 1.46)
Model 2, HR (95%CI)	1 (reference)	1.08 (0.99, 1.19)	1.06 (0.97, 1.16)	1.14 (1.04, 1.25)	1.31 (1.19, 1.44)
Model 3, HR (95%CI)	1 (reference)	1.08 (0.99, 1.19)	1.06 (0.97, 1.16)	1.14 (1.04, 1.25)	1.31 (1.19, 1.44)
Lp(a) concentrations					
	Quintile 1	Quintile 2	Quintile 3	Quintile 4	Quintile 5
Cases/ Total	1,580/64,104	1,625/64,108	1,627/64,092	1,668/64,104	1,665/64,098
IR, per 1,000 person-years	1.81	1.86	1.87	1.92	1.91
Model 1, HR (95%CI)	1 (reference)	1.03 (0.96, 1.11)	1.01 (0.95, 1.09)	1.03 (0.96, 1.11)	1.07 (1.00, 1.14)
Model 2, HR (95%CI)	1 (reference)	1.04 (0.97, 1.11)	1.02 (0.95, 1.09)	1.04 (0.97, 1.11)	1.07 (1.00, 1.15)
Model 3, HR (95%CI)	1 (reference)	1.04 (0.97, 1.11)	1.02 (0.95, 1.09)	1.04 (0.97, 1.11)	1.07 (1.00, 1.15)

Model 1 was adjusted for age and sex.

Model 2 was further adjusted for TDI, ethnicity, current smoking status, physical activity, systolic blood pressure, BMI, creatinine, and use of antihypertensive and lipid-lowering medications.

Model 3 was additionally adjusted for the other two biomarkers (TyG index, hs-CRP, and Lp(a), as applicable) based on Model 2.

Abbreviations: TyG, triglyceride–glucose; hs-CRP, high-sensitivity C-reactive protein; Lp(a), lipoprotein(a); IR, incidence rate; HR, hazard ratio; CI, confidence interval; ASCVD, atherosclerotic cardiovascular disease; CHD, coronary heart disease; TDI, Townsend deprivation index; BMI, body mass index.

Table S5. Associations of biomarker combination score defined by TyG, hs-CRP, and Lp(a) with incident ASCVD and its subtypes

Panel A. ASCVD

Score	0	1	2	3
Cases/ Total	13,601/167,399	13,403/117,152	5,082/32,634	657/3,321
IR, per 1,000 person-years	6.08	8.78	12.30	16.10
Model 1, HR (95%CI)	1 (reference)	1.20 (1.17, 1.23)	1.54 (1.48, 1.60)	2.04 (1.85, 2.24)
Model 2, HR (95%CI)	1 (reference)	1.18 (1.15, 1.21)	1.48 (1.42, 1.53)	1.85 (1.68, 2.03)

Panel B. CHD

Score	0	1	2	3
Cases/ Total	10,504/167,399	10,838/117,152	4,270/32,634	564/3,321
IR, per 1,000 person-years	4.67	7.04	10.20	13.60
Model 1, HR (95%CI)	1 (reference)	1.23 (1.19, 1.26)	1.64 (1.58, 1.71)	2.25 (2.03, 2.50)
Model 2, HR (95%CI)	1 (reference)	1.21 (1.18, 1.24)	1.56 (1.50, 1.63)	2.00 (1.81, 2.22)

Panel C. Stroke

Score	0	1	2	3
Cases/ Total	3,702/167,399	3,237/117,152	1,094/32,634	132/3,321
IR, per 1,000 person-years	1.61	2.04	2.51	2.99
Model 1, HR (95%CI)	1 (reference)	1.10 (1.05, 1.16)	1.22 (1.13, 1.32)	1.35 (1.10, 1.66)
Model 2, HR (95%CI)	1 (reference)	1.11 (1.06, 1.17)	1.22 (1.13, 1.32)	1.31 (1.07, 1.61)

Model 1 was adjusted for age and sex.

Model 2 was further adjusted for TDI, ethnicity, current smoking status, physical activity, systolic blood pressure, BMI, creatinine, and use of antihypertensive and lipid-lowering medications.

Abbreviations: TyG, triglyceride–glucose; hs-CRP, high-sensitivity C-reactive protein; Lp(a), lipoprotein(a); IR, incidence rate; HR, hazard ratio; CI, confidence interval; ASCVD, atherosclerotic cardiovascular disease; CHD, coronary heart disease; TDI, Townsend deprivation index; BMI, body mass index.

Table S6. Associations of biomarker combination patterns defined by TyG, hs-CRP, and Lp(a) with incident ASCVD and its subtypes

Panel A. ASCVD

	Group 1	Group 2	Group 3	Group 4	Group 5	Group 6	Group 7	Group 8
Cases/ Total	13,601/167,399	5,017/37,734	4,380/36,611	4,006/42,807	2,447/14,664	1,315/8,382	1,320/9,588	657/3,321
IR, per 1,000 person-years	6.08	10.30	9.31	7.04	13.30	12.30	10.80	16.10
Model 1, HR (95%CI)	1 (reference)	1.21 (1.16, 1.25)	1.25 (1.20, 1.30)	1.16 (1.11, 1.20)	1.57 (1.48, 1.66)	1.54 (1.43, 1.65)	1.46 (1.36, 1.57)	2.00 (1.80, 2.23)
Model 2, HR (95%CI)	1 (reference)	1.18 (1.14, 1.22)	1.26 (1.21, 1.31)	1.15 (1.11, 1.20)	1.51 (1.42, 1.60)	1.48 (1.37, 1.59)	1.47 (1.37, 1.58)	1.90 (1.71, 2.11)

Panel B. CHD

Group	Group 1	Group 2	Group 3	Group 4	Group 5	Group 6	Group 7	Group 8
Cases/ Total	10,504/167,399	4,140/37,734	3,499/36,611	3,199/42,807	2,039/14,664	1,114/8,382	1,117/9,588	564/3,321
IR, per 1,000 person-years	4.67	8.42	7.36	5.58	11.00	10.30	9.06	13.60
Model 1, HR (95%CI)	1 (reference)	1.25 (1.20, 1.30)	1.25 (1.20, 1.31)	1.18 (1.13, 1.24)	1.66 (1.56, 1.78)	1.62 (1.50, 1.75)	1.57 (1.46, 1.70)	2.21 (1.97, 2.48)
Model 2, HR (95%CI)	1 (reference)	1.21 (1.16, 1.26)	1.27 (1.21, 1.33)	1.18 (1.13, 1.23)	1.58 (1.48, 1.69)	1.54 (1.42, 1.67)	1.58 (1.46, 1.71)	2.08 (1.85, 2.33)

Panel C. Stroke

	Group 1	Group 2	Group 3	Group 4	Group 5	Group 6	Group 7	Group 8
Cases/ Total	3,702/167,399	1,114/37,734	1,142/36,611	981/42,807	542/14,664	286/8,382	266/9,588	132/3,321
IR, per 1,000 person-years	1.61	2.18	2.34	1.67	2.78	2.53	2.07	2.99
Model 1,	1 (reference)	1.07 (0.99, 1.15)	1.27 (1.17, 1.37)	1.06 (0.98, 1.15)	1.27 (1.12, 1.43)	1.32 (1.13, 1.53)	1.09 (0.93, 1.27)	1.31 (1.03, 1.66)

HR (95%CI)								
Model 2, HR (95%CI)	1 (reference)	1.08 (1.00, 1.16)	1.26 (1.17, 1.36)	1.07 (0.99, 1.16)	1.27 (1.13, 1.44)	1.32 (1.13, 1.53)	1.09 (0.93, 1.27)	1.31 (1.03, 1.66)

Biomarker combination patterns: (1) TyG, hs-CRP, and Lp(a) all below the fifth quintile; (2) TyG in the fifth quintile only; (3) hs-CRP in the fifth quintile only; (4) Lp(a) in the fifth quintile only; (5) TyG and hs-CRP in the fifth quintile; (6) TyG and Lp(a) in the fifth quintile; (7) hs-CRP and Lp(a) in the fifth quintile; and (8) TyG, hs-CRP, and Lp(a) all in the fifth quintile.

Model 1 was adjusted for age and sex.

Model 2 was further adjusted for TDI, ethnicity, current smoking status, physical activity, systolic blood pressure, BMI, creatinine, and use of antihypertensive and lipid-lowering medications.

Abbreviations: TyG, triglyceride–glucose; hs-CRP, high-sensitivity C-reactive protein; Lp(a), lipoprotein(a); IR, incidence rate; HR, hazard ratio; CI, confidence interval; ASCVD, atherosclerotic cardiovascular disease; CHD, coronary heart disease; TDI, Townsend deprivation index; BMI, body mass index.

Table S7. Stratified analyses of individual and joint biomarker associations with incident ASCVD by sex and age group

Panel A. TyG concentrations

	Quintile 1	Quintile 2	Quintile 3	Quintile 4	Quintile 5	Nominal *P* for Interaction
Sex						0.001567
Men						
Cases/Total	2,087/19,552	2,941/24,227	3,806/28,239	4,628/32,359	6,480/38,629	
IR, per 1,000 person-years	8.14	9.38	10.50	11.20	13.30	
HR (95% CI)	1.00 (reference)	1.02 (0.97, 1.08)	1.09 (1.03, 1.15)	1.11 (1.05, 1.17)	1.28 (1.21, 1.34)	
Women						
Cases/Total	1,897/44,549	2,376/39,874	2,709/35,863	2,863/31,742	2,956/25,472	
IR, per 1,000 person-years	3.10	4.41	5.65	6.81	8.92	
HR (95% CI)	1.00 (reference)	1.06 (1.00, 1.13)	1.15 (1.08, 1.22)	1.23 (1.15, 1.30)	1.41 (1.33, 1.50)	
Age						< 2.2e-16
>=60Y						
Cases/Total	2,347/18,332	3,376/25,738	4,209/28,819	4,807/30,398	5,535/29,242	
IR, per 1,000 person-years	9.97	10.30	11.60	12.60	15.40	
HR (95% CI)	1.00 (reference)	0.97 (0.92, 1.02)	1.02 (0.97, 1.07)	1.04 (0.98, 1.09)	1.16 (1.10, 1.22)	
<60Y						
Cases/Total	1,637/45,769	1,941/38,363	2,306/35,283	2,684/33,703	3,901/34,859	
IR, per 1,000 person-years	2.59	3.70	4.83	5.92	8.48	
HR (95% CI)	1.00 (reference)	1.14 (1.07, 1.22)	1.27 (1.19, 1.36)	1.37 (1.28, 1.46)	1.63 (1.53, 1.74)	

Panel B. hs-CRP concentrations

	Quintile 1	Quintile 2	Quintile 3	Quintile 4	Quintile 5	Nominal *P* for Interaction
Sex						0.9454
Men						
Cases/Total	2,575/27,280	3,688/30,894	4,284/30,499	4,617/28,972	4,778/25,361	
IR, per 1,000 person-years	7.10	9.13	10.90	12.60	15.50	
HR (95% CI)	1.00 (reference)	1.09 (1.03, 1.14)	1.18 (1.12, 1.24)	1.25 (1.19, 1.32)	1.44 (1.37, 1.52)	
Women						
Cases/Total	1,580/36,346	1,967/33,852	2,313/33,372	2,915/35,107	4,026/38,823	
IR, per 1,000 person-years	3.18	4.28	5.15	6.24	7.92	
HR (95% CI)	1.00 (reference)	1.07 (1.00, 1.15)	1.11 (1.04, 1.19)	1.21 (1.13, 1.29)	1.39 (1.30, 1.48)	
Age						4.682e-10
>=60Y						
Cases/Total	2,372/20,165	3,415/25,595	4,211/27,860	4,793/29,423	5,483/29,486	
IR, per 1,000 person-years	9.09	10.40	11.90	13.00	15.30	
HR (95% CI)	1.00 (reference)	1.03 (0.97, 1.08)	1.12 (1.06, 1.18)	1.17 (1.11, 1.23)	1.33 (1.26, 1.40)	
<60Y						
Cases/Total	1,783/43,461	2,240/39,151	2,386/36,011	2,739/34,656	3,321/34,698	
IR, per 1,000 person-years	2.98	4.19	4.88	5.88	7.23	
HR (95% CI)	1.00 (reference)	1.15 (1.08, 1.22)	1.18 (1.11, 1.26)	1.31 (1.23, 1.40)	1.55 (1.45, 1.65)	

Panel C. Lp(a) concentrations

	Quintile 1	Quintile 2	Quintile 3	Quintile 4	Quintile 5	Nominal *P* for Interaction
Sex						0.03218
Men						
Cases/Total	3,944/30,386	3,961/29,803	3,696/27,856	3,837/26,463	4,504/28,498	
IR, per 1,000 person-years	10.10	10.30	10.30	11.40	12.40	
HR (95% CI)	1.00 (reference)	1.05 (1.00, 1.09)	1.04 (0.99, 1.09)	1.13 (1.08, 1.18)	1.29 (1.23, 1.34)	
Women						
Cases/Total	2,270/33,718	2,292/34,305	2,547/36,236	2,898/37,641	2,794/35,600	
IR, per 1,000 person-years	5.00	4.96	5.24	5.76	5.87	
HR (95% CI)	1.00 (reference)	0.99 (0.94, 1.05)	0.99 (0.94, 1.05)	1.07 (1.01, 1.13)	1.15 (1.09, 1.22)	
Age						0.7376
>=60Y						
Cases/Total	3,881/25,772	3,812/25,900	3,936/27,509	4,223/27,475	4,422/25,873	
IR, per 1,000 person-years	12.00	11.60	11.30	12.20	13.70	
HR (95% CI)	1.00 (reference)	0.99 (0.95, 1.04)	0.99 (0.95, 1.04)	1.08 (1.04, 1.13)	1.20 (1.15, 1.25)	
<60Y						
Cases/Total	2,333/38,332	2,441/38,208	2,307/36,583	2,512/36,629	2,876/38,225	
IR, per 1,000 person-years	4.48	4.71	4.64	5.08	5.57	
HR (95% CI)	1.00 (reference)	1.08 (1.02, 1.14)	1.07 (1.01, 1.14)	1.15 (1.09, 1.22)	1.29 (1.22, 1.36)	

Panel D. Biomarker combination patterns defined by TyG, hs-CRP, and Lp(a)

	Group 1	Group 2	Group 3	Group 4	Group 5	Group 6	Group 7	Group 8	Nominal *P* for Interaction
Sex									0.002407
Men									
Cases/Total	8,038/69,405	3,717/24,684	2,273/13,523	2,451/18,044	1,410/6,896	958/5,512	700/3,405	395/1,537	
IR, per 1,000 person-years	8.87	11.80	13.70	10.50	16.80	13.70	17.00	21.70	
HR (95% CI)	1.00 (reference)	1.19 (1.15, 1.24)	1.25 (1.20, 1.32)	1.20 (1.15, 1.26)	1.44 (1.35, 1.52)	1.43 (1.34, 1.53)	1.57 (1.45, 1.70)	1.89 (1.71, 2.10)	
Women									
Cases/Total	5,563/97,994	1,300/13,050	2,107/23,088	1,555/24,763	1,037/7,768	357/2,870	620/6,183	262/1,784	
IR, per 1,000 person-years	4.18	7.58	6.92	4.64	10.40	9.58	7.65	11.50	
HR (95% CI)	1.00 (reference)	1.21 (1.14, 1.29)	1.25 (1.19, 1.32)	1.10 (1.04, 1.16)	1.51 (1.41, 1.63)	1.58 (1.42, 1.76)	1.38 (1.27, 1.51)	1.69 (1.49, 1.92)	
Age									< 2.2e-16
>=60Y									
Cases/Total	8,628/65,864	2,955/17,108	2,813/16,748	2,463/16,423	1,456/6,936	745/3,648	835/4,252	379/1,550	
IR, per 1,000 person-years	10.20	13.90	13.70	11.80	17.50	16.60	16.30	20.90	
HR (95% CI)	1.00 (reference)	1.12 (1.07, 1.17)	1.19 (1.14, 1.25)	1.15 (1.10, 1.20)	1.37 (1.29, 1.45)	1.36 (1.26, 1.47)	1.41 (1.31, 1.52)	1.64 (1.48, 1.82)	
<60Y									
Cases/Total	4,973/101,535	2,062/20,626	1,567/19,863	1,543/26,384	991/7,728	570/4,734	485/5,336	278/1,771	
IR, per 1,000 person-years	3.57	7.51	5.91	4.28	9.86	9.14	6.85	12.20	

HR (95% CI)	1.00 (reference)	1.34 (1.27, 1.41)	1.36 (1.28, 1.44)	1.18 (1.11, 1.25)	1.64 (1.53, 1.77)	1.63 (1.49, 1.78)	1.58 (1.44, 1.74)	2.10 (1.86, 2.38)

Panel A-C. Model stratified by sex was adjusted for age, TDI, ethnicity, current smoking status, physical activity, systolic blood pressure, BMI, creatinine, use of antihypertensive and lipid-lowering medications, and the other two biomarkers (TyG index, hs-CRP, and Lp(a), as applicable). Model stratified by age was adjusted for age, sex, TDI, ethnicity, current smoking status, physical activity, systolic blood pressure, BMI, creatinine, use of antihypertensive and lipid-lowering medications, and the other two biomarkers (TyG index, hs-CRP, and Lp(a), as applicable).

Panel D. Biomarker combination patterns: (1) TyG, hs-CRP, and Lp(a) all below the fifth quintile; (2) TyG in the fifth quintile only; (3) hs-CRP in the fifth quintile only; (4) Lp(a) in the fifth quintile only; (5) TyG and hs-CRP in the fifth quintile; (6) TyG and Lp(a) in the fifth quintile; (7) hs-CRP and Lp(a) in the fifth quintile; and (8) TyG, hs-CRP, and Lp(a) all in the fifth quintile. Model stratified by sex was adjusted for age, TDI, ethnicity, current smoking status, physical activity, systolic blood pressure, BMI, creatinine, and use of antihypertensive and lipid-lowering medications. Model stratified by age was adjusted for age, sex, TDI, ethnicity, current smoking status, physical activity, systolic blood pressure, BMI, creatinine, and use of antihypertensive and lipid-lowering medications.

Abbreviations: TyG, triglyceride–glucose; hs-CRP, high-sensitivity C-reactive protein; Lp(a), lipoprotein(a); IR, incidence rate; HR, hazard ratio; CI, confidence interval; ASCVD, atherosclerotic cardiovascular disease; CHD, coronary heart disease; TDI, Townsend deprivation index; BMI, body mass index.

Table S8. Sensitivity analysisof individual and joint biomarker associations with incident ASCVD

Panel A. TyG concentrations

	Quintile 1	Quintile 2	Quintile 3	Quintile 4	Quintile 5
Excluded participants with cancer (N=28,347)					
Cases/Total	3,578/59,073	4,740/58,436	5,800/58,194	6,631/58,208	8,484/58,248
IR, per 1,000 person-years	4.46	6.08	7.55	8.70	11.30
HR (95% CI)	1.00 (reference)	1.06 (1.01, 1.10)	1.14 (1.09, 1.19)	1.18 (1.13, 1.23)	1.38 (1.33, 1.44)
2-year lagged analysis (N=4,445)					
Cases/Total	3,633/63,576	4,827/63,389	5,831/63,185	6,769/63,138	8,366/62,773
IR, per 1,000 person-years	4.19	5.67	6.93	8.12	10.20
HR (95% CI)	1.00 (reference)	1.05 (1.01, 1.10)	1.13 (1.08, 1.17)	1.18 (1.13, 1.23)	1.35 (1.29, 1.40)
Follow-up interval-specific sensitivity analyses					
Year 0 to 5					
Cases/Total	1,087/64,101	1,476/64,101	1,905/64,102	2,161/64,101	2,977/64,101
IR, per 1,000 person-years	3.43	4.68	6.06	6.89	9.56
HR (95% CI)	1.00 (reference)	1.04 (0.96, 1.12)	1.16 (1.07, 1.25)	1.17 (1.08, 1.26)	1.43 (1.33, 1.54)
Year 5 to 10					
Cases/Total	1,489/62,340	2,075/61,764	2,500/61,322	2,905/61,043	3,569/60,153
IR, per 1,000 person-years	4.87	6.90	8.40	9.85	12.40
HR (95% CI)	1.00 (reference)	1.10 (1.03, 1.18)	1.17 (1.09, 1.25)	1.23 (1.15, 1.31)	1.39 (1.30, 1.48)
Year >10					
Cases/Total	1,408/59,831	1,766/58,444	2,110/57,545	2,425/56,714	2,890/55,014
IR, per 1,000	5.73	7.47	9.16	10.70	13.20

person-years					
HR (95% CI)	1.00 (reference)	1.01 (0.95, 1.09)	1.08 (1.01, 1.16)	1.14 (1.07, 1.22)	1.27 (1.18, 1.36)
Fine-Gray					
Cases/Total	3,984/64,101	5,317/64,101	6,515/64,102	7,491/64,101	9,436/64,101
IR, per 1,000 person-years	4.59	6.24	7.74	8.97	11.50
sHR (95% CI)	1.00 (reference)	1.05 (1.01, 1.10)	1.14 (1.10, 1.19)	1.18 (1.14, 1.23)	1.36 (1.31, 1.42)

Panel B. hs-CRP concentrations

	Quintile 1	Quintile 2	Quintile 3	Quintile 4	Quintile 5
Excluded participants with cancer (N=28,347)					
Cases/Total	3,742/58,690	5,123/59,478	5,889/58,309	6,723/58,131	7,756/57,551
IR, per 1,000 person-years	4.71	6.44	7.63	8.84	10.50
HR (95% CI)	1.00 (reference)	1.09 (1.05, 1.14)	1.16 (1.11, 1.21)	1.25 (1.20, 1.30)	1.44 (1.38, 1.50)
2-year lagged analysis (N=4,445)					
Cases/Total	3,811/63,171	5,123/64,072	5,934/63,027	6,731/63,073	7,827/62,718
IR, per 1,000 person-years	4.43	5.94	7.05	8.08	9.59
HR (95% CI)	1.00 (reference)	1.07 (1.03, 1.12)	1.14 (1.10, 1.19)	1.22 (1.17, 1.28)	1.41 (1.35, 1.47)
Follow-up interval-specific sensitivity analyses					
Year 0 to 5					
Cases/Total	1,085/63,626	1,599/64,746	1,870/63,871	2,268/64,079	2,784/64,184
IR, per 1,000 person-years	3.45	5.01	5.96	7.24	8.95
HR (95% CI)	1.00 (reference)	1.13 (1.05, 1.23)	1.19 (1.10, 1.29)	1.33 (1.23, 1.44)	1.56 (1.45, 1.69)
Year 5 to 10					

Cases/Total	1,578/61,981	2,157/62,501	2,575/61,281	2,863/60,893	3,365/59,966
IR, per 1,000 person-years	5.19	7.07	8.66	9.75	11.70
HR (95% CI)	1.00 (reference)	1.09 (1.02, 1.16)	1.19 (1.12, 1.27)	1.25 (1.17, 1.33)	1.45 (1.35, 1.54)
Year >10					
Cases/Total	1,492/59,434	1,899/59,283	2,152/57,502	2,401/56,533	2,655/54,796
IR, per 1,000 person-years	6.18	7.93	9.29	10.60	12.10
HR (95% CI)	1.00 (reference)	1.04 (0.97, 1.11)	1.09 (1.02, 1.17)	1.16 (1.08, 1.24)	1.28 (1.19, 1.37)
Fine-Gray					
Cases/Total	4,155/63,626	5,655/64,746	6,597/63,871	7,532/64,079	8,804/64,184
IR, per 1,000 person-years	4.83	6.55	7.83	9.03	10.80
sHR (95% CI)	1.00 (reference)	1.10 (1.06, 1.15)	1.18 (1.14, 1.23)	1.26 (1.21, 1.31)	1.39 (1.32, 1.45)

Panel C. hs-CRP concentrations

	Quintile 1	Quintile 2	Quintile 3	Quintile 4	Quintile 5
Excluded participants with cancer (N=28,347)					
Cases/Total	5,565/58,508	5,579/58,490	5,568/58,283	5,996/58,245	6,525/58,633
IR, per 1,000 person-years	7.18	7.20	7.21	7.82	8.45
HR (95% CI)	1.00 (reference)	1.02 (0.99, 1.06)	1.02 (0.98, 1.06)	1.10 (1.06, 1.14)	1.22 (1.18, 1.27)
2-year lagged analysis (N=4,445)					
Cases/Total	5,587/63,255	5,642/63,274	5,625/63,235	6,020/63,145	6,552/63,152
IR, per 1,000 person-years	6.62	6.68	6.66	7.17	7.81
HR (95% CI)	1.00 (reference)	1.03 (0.99, 1.07)	1.03 (0.99, 1.06)	1.10 (1.06, 1.15)	1.23 (1.19, 1.28)

Follow-up interval-specific sensitivity analyses					
Year 0 to 5					
Cases/Total	1,768/64,104	1,826/64,108	1,837/64,092	2,034/64,104	2,141/64,098
IR, per 1,000 person-years	5.62	5.80	5.84	6.48	6.82
HR (95% CI)	1.00 (reference)	1.06 (1.00, 1.13)	1.07 (1.00, 1.14)	1.18 (1.11, 1.26)	1.27 (1.20, 1.36)
Year 5 to 10					
Cases/Total	2,381/61,440	2,384/61,462	2,376/61,374	2,597/61,205	2,800/61,141
IR, per 1,000 person-years	7.99	7.99	7.98	8.76	9.46
HR (95% CI)	1.00 (reference)	1.02 (0.97, 1.08)	1.02 (0.96, 1.08)	1.12 (1.05, 1.18)	1.24 (1.17, 1.30)
Year >10					
Cases/Total	2,065/57,679	2,043/57,757	2,030/57,699	2,104/57,274	2,357/57,139
IR, per 1,000 person-years	8.88	8.76	8.73	9.15	10.30
HR (95% CI)	1.00 (reference)	1.00 (0.94, 1.07)	0.99 (0.93, 1.06)	1.05 (0.98, 1.11)	1.20 (1.13, 1.28)
Fine-Gray					
Cases/Total	6,214/64,104	6,253/64,108	6,243/64,092	6,735/64,104	7,298/64,098
IR, per 1,000 person-years	7.35	7.39	7.39	8.01	8.69
sHR (95% CI)	1.00 (reference)	1.03 (1.00, 1.07)	1.03 (0.99, 1.07)	1.12 (1.08, 1.16)	1.24 (1.20, 1.29)

Panel D. Biomarker combination patterns defined by TyG, hs-CRP, and Lp(a)

	Group 1	Group 2	Group 3	Group 4	Group 5	Group 6	Group 7	Group 8
Excluded participants with cancer (N=28,347)								
Cases/Total	12,141/153,027	4,546/34,555	3,855/32,870	3,597/39,373	2,166/13,074	1,193/7,653	1,156/8,641	579/2,966
IR, per 1,000	5.92	10.10	9.06	6.84	13.10	12.20	10.40	15.70

person-years								
HR (95% CI)	1.00 (reference)	1.23 (1.18, 1.27)	1.27 (1.22, 1.32)	1.16 (1.12, 1.21)	1.51 (1.44, 1.59)	1.50 (1.42, 1.60)	1.47 (1.38, 1.56)	1.85 (1.70, 2.02)
2-year lagged analysis (N=4,445)								
Cases/Total	12,343/165,735	4,455/37,053	3,922/35,855	3,617/42,322	2,154/14,266	1,184/8,233	1,178/9,376	573/3,221
IR, per 1,000 person-years	5.53	9.16	8.35	6.36	11.80	11.10	9.66	14.00
HR (95% CI)	1.00 (reference)	1.19 (1.15, 1.24)	1.25 (1.21, 1.30)	1.16 (1.11, 1.20)	1.46 (1.40, 1.54)	1.48 (1.40, 1.58)	1.47 (1.38, 1.56)	1.79 (1.64, 1.95)
Follow-up interval-specific sensitivity analyses								
Year 0 to 5								
Cases/Total	3,768/167,399	1,561/37,734	1,341/36,611	1,084/42,807	795/14,664	409/8,382	436/9,588	212/3,321
IR, per 1,000 person-years	4.57	8.49	7.53	5.15	11.20	10.00	9.38	13.30
HR (95% CI)	1.00 (reference)	1.29 (1.22, 1.37)	1.32 (1.23, 1.40)	1.13 (1.05, 1.21)	1.59 (1.47, 1.73)	1.56 (1.41, 1.73)	1.64 (1.49, 1.82)	1.91 (1.66, 2.20)
Year 5 to 10								
Cases/Total	5,218/161,838	1,924/35,669	1,690/34,401	1,547/41,281	906/13,573	484/7,868	514/8,949	255/3,043
IR, per 1,000 person-years	6.61	11.20	10.20	7.69	14.00	12.80	12.00	17.80
HR (95% CI)	1.00 (reference)	1.21 (1.15, 1.28)	1.27 (1.20, 1.34)	1.17 (1.10, 1.24)	1.44 (1.33, 1.55)	1.43 (1.30, 1.57)	1.50 (1.36, 1.64)	1.87 (1.64, 2.12)
Year >10								
Cases/Total	4,615/153,592	1,532/32,916	1,349/31,695	1,375/39,045	746/12,206	422/7,199	370/8,202	190/2,693
IR, per 1,000 person-years	7.43	11.60	10.60	8.72	15.40	14.70	11.30	17.90
HR (95% CI)	1.00 (reference)	1.13 (1.06, 1.20)	1.18 (1.11, 1.25)	1.18 (1.11, 1.25)	1.41 (1.30, 1.53)	1.47 (1.33, 1.62)	1.28 (1.15, 1.43)	1.66 (1.44, 1.93)
Sensitivity analysis using alternative biomarker thresholds for incident ASCVD*								
Cases/Total	6,542/98,636	8,671/78,566	3,614/36,016	1,432/18,399	8,323/58,805	1,643/12,970	828/7,186	1,690/9,928
IR, per 1,000 person-years	4.91	8.41	7.68	5.79	11.10	9.72	8.89	13.60

HR (95% CI)	1.00 (reference)	1.17 (1.14, 1.21)	1.24 (1.19, 1.29)	1.14 (1.08, 1.21)	1.41 (1.36, 1.46)	1.38 (1.31, 1.46)	1.42 (1.32, 1.53)	1.76 (1.66, 1.86)
Fine-Gray								
Cases/Total	13,601/167,399	5,017/37,734	4,380/36,611	4,006/42,807	2,447/14,664	1,315/8,382	1,320/9,588	657/3,321
IR, per 1,000 person-years	6.08	10.30	9.31	7.04	13.30	12.30	10.80	16.10
sHR (95% CI)	1.00 (reference)	1.01 (0.97, 1.06)	1.15 (1.10, 1.19)	1.17 (1.13, 1.21)	1.15 (1.09, 1.22)	1.24 (1.16, 1.32)	1.36 (1.28, 1.45)	1.42 (1.30, 1.55)

Panel D. Biomarker combination patterns: (1) TyG, hs-CRP, and Lp(a) all below the fifth quintile; (2) TyG in the fifth quintile only; (3) hs-CRP in the fifth quintile only; (4) Lp(a) in the fifth quintile only; (5) TyG and hs-CRP in the fifth quintile; (6) TyG and Lp(a) in the fifth quintile; (7) hs-CRP and Lp(a) in the fifth quintile; and (8) TyG, hs-CRP, and Lp(a) all in the fifth quintile.

* (1) TyG, hs-CRP, and Lp(a) all below the thresholds; (2) TyG over the threshold only; (3) hs-CRP over the thresholds only; (4) Lp(a) over the thresholds only; (5) TyG and hs-CRP over the thresholds; (6) TyG and Lp(a) over the thresholds; (7) hs-CRP and Lp(a) over the thresholds ; and (8) TyG, hs-CRP, and Lp(a) all over the thresholds.

Panel A-C. Model was adjusted for age, sex, TDI, ethnicity, current smoking status, physical activity, systolic blood pressure, BMI, creatinine, use of antihypertensive and lipid-lowering medications, and the other two biomarkers (TyG index, hs-CRP, and Lp(a), as applicable);

Panel D. Model was adjusted for age, sex, TDI, ethnicity, current smoking status, physical activity, systolic blood pressure, BMI, creatinine, use of antihypertensive and lipid-lowering medications.

Abbreviations: TyG, triglyceride–glucose; hs-CRP, high-sensitivity C-reactive protein; Lp(a), lipoprotein(a); IR, incidence rate; HR, hazard ratio; CI, confidence interval; ASCVD, atherosclerotic cardiovascular disease; TDI, Townsend deprivation index; BMI, body mass index; sHR, subdistribution hazard ratio.

Table S9. Incremental predictive performance of TyG, hs-CRP, and Lp(a), individually and jointly, for 10-year incident ASCVD

Model	C-statistic	ΔC-statistic	10-year AUC	ΔAUC	Brier score	Continuous NRI, %	IDI, %
Clinical model	0.712	Reference	0.729	Reference	0.062	Reference	Reference
Clinical + TyG	0.714	0.002	0.732	0.003	0.062	13.52	0.07
Clinical + hs-CRP	0.715	0.003	0.732	0.003	0.062	13.47	0.16
Clinical + Lp(a)	0.713	0.001	0.730	0.001	0.062	6.96	0.05
Clinical + TyG + hs-CRP + Lp(a)	0.717	0.005	0.735	0.006	0.062	18.65	0.27

Abbreviations: TyG, triglyceride–glucose; hs-CRP, high-sensitivity C-reactive protein; Lp(a), lipoprotein(a); ASCVD, atherosclerotic cardiovascular disease; AUC, area under the receiver operating characteristic curve; NRI, net reclassification improvement; IDI, integrated discrimination improvement.

Figure S1. Scaled Schoenfeld residual plots for exposure terms in the Cox models

Panel A. Group of TyG concentration

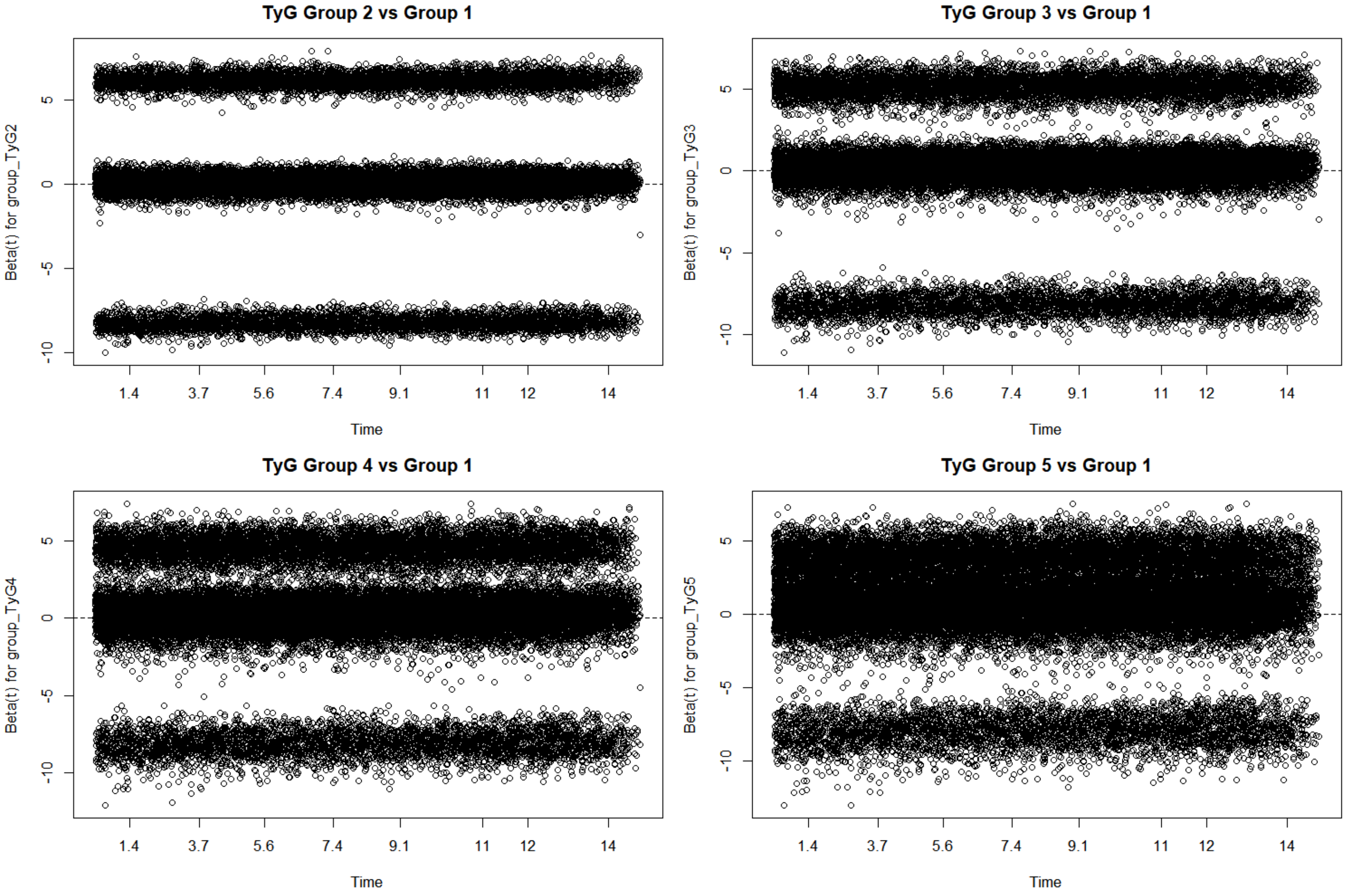

Panel B. Group of hs-CRP concentration

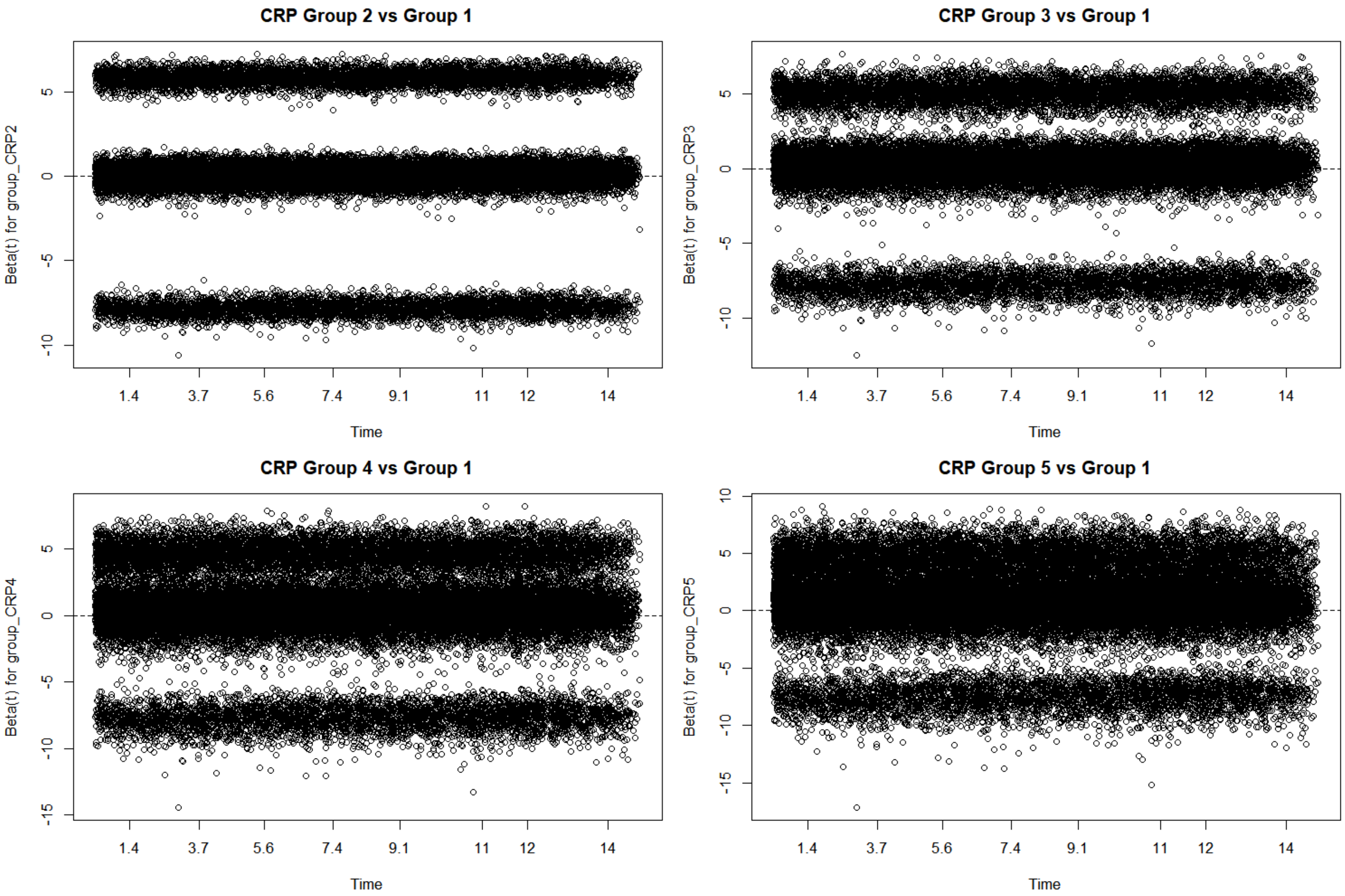

Panel C. Group of Lp(a) concentration

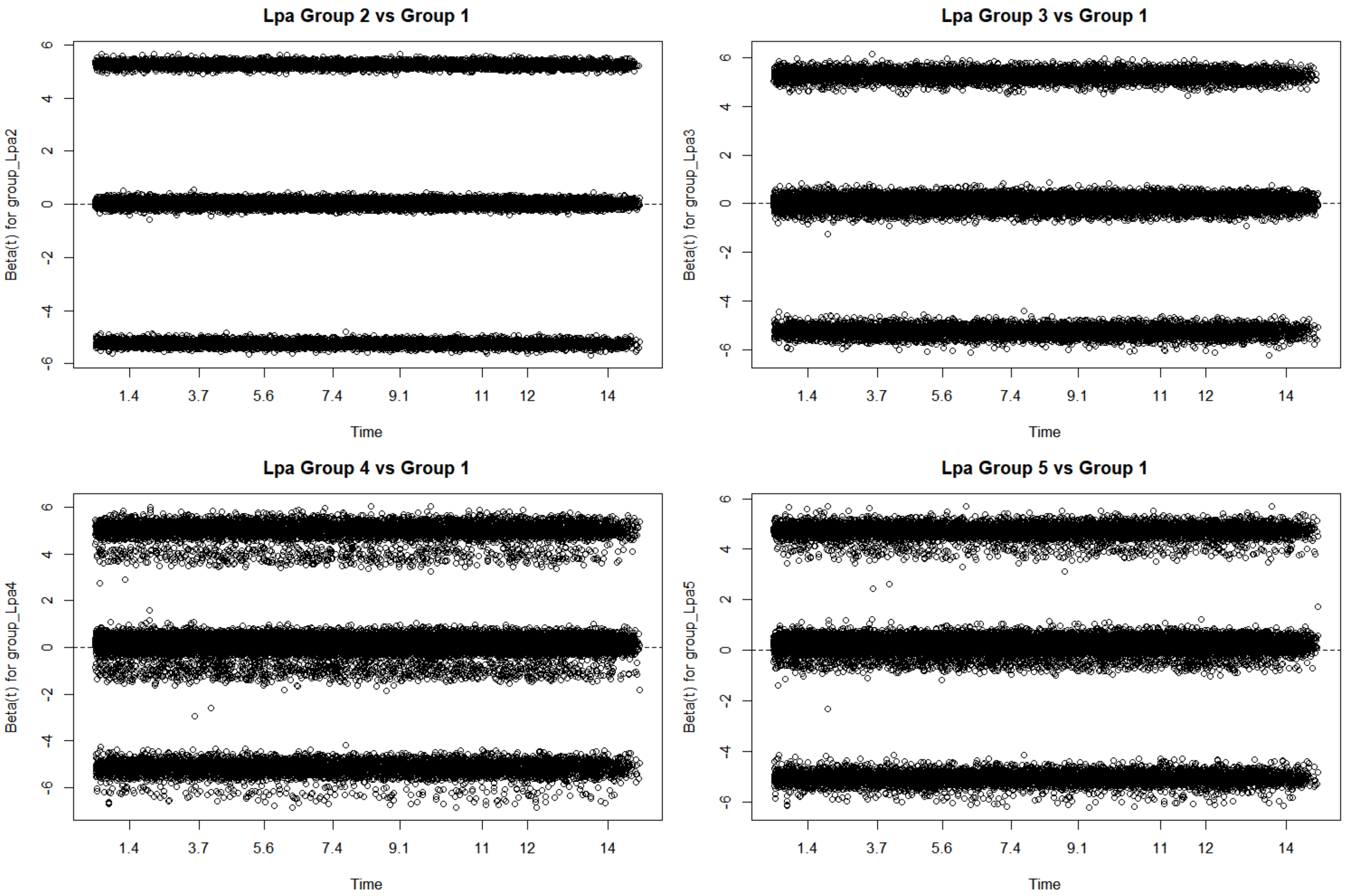

Panel D. Group of biomarker combination patterns defined by TyG, hs-CRP, and Lp(a)

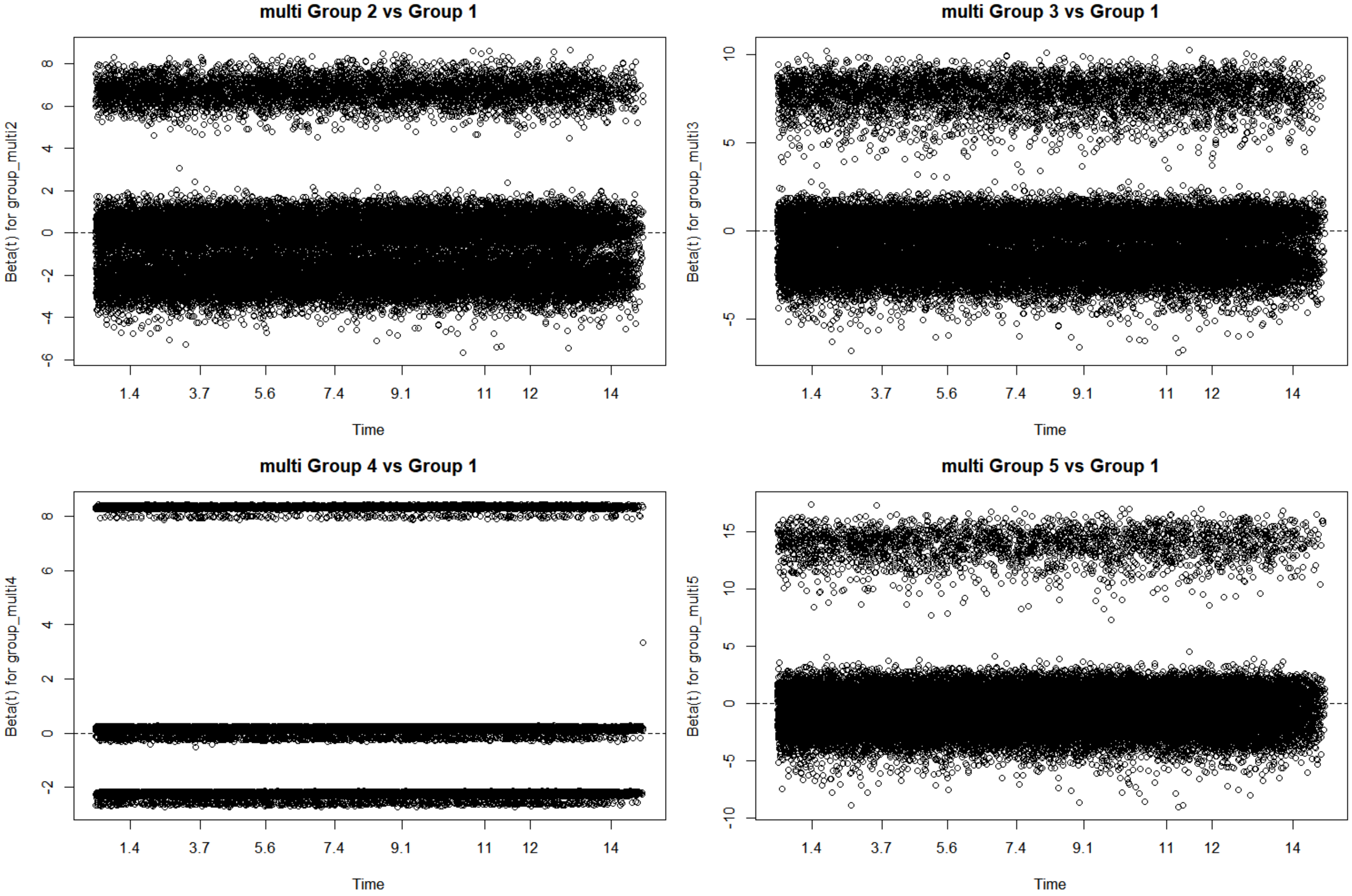

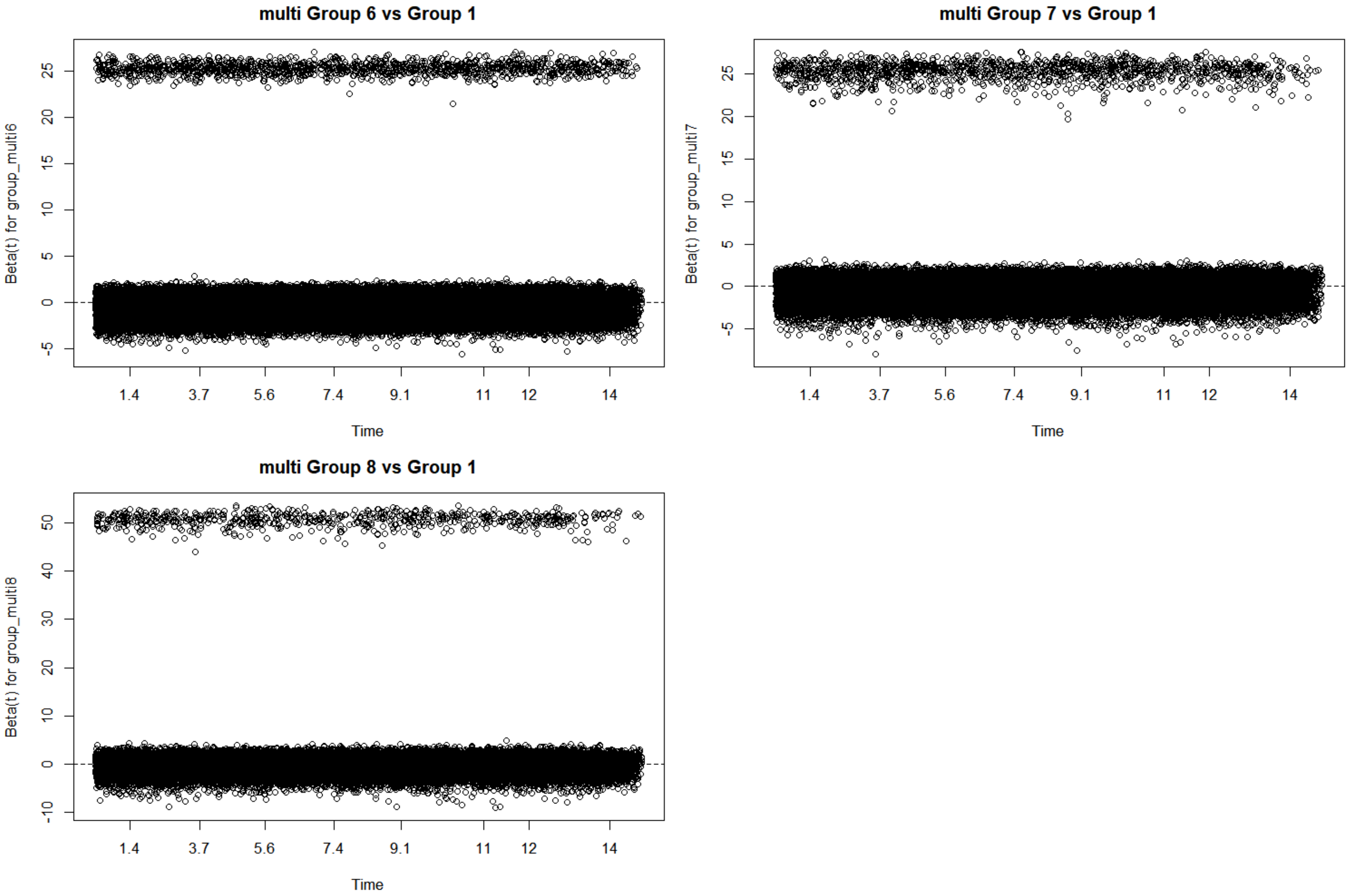

Abbreviations: TyG, triglyceride–glucose; hs-CRP, high-sensitivity C-reactive protein; Lp(a), lipoprotein(a).
